# HaDeX2: multi-dimensional analysis of hydrogen–deuterium exchange mass spectrometry data

**DOI:** 10.1093/bioinformatics/btag128

**Published:** 2026-03-16

**Authors:** Weronika Puchała, Krystyna Grzesiak, Dominik Rafacz, Michał Kistowski, Jochem H Smit, Julien Marcoux, Michał Dadlez, Michał Burdukiewicz

**Affiliations:** Institute of Biochemistry and Biophysics, Polish Academy of Sciences, 02-106 Warsaw, Poland; Faculty of Mathematics and Computer Science, University of Wrocław, 50-383 Wrocław, Poland; Clinical Research Centre, Medical University of Białystok, 15-089 Białystok, Poland; Faculty of Mathematics and Information Science, Warsaw University of Technology, 00-666 Warsaw, Poland; Institute of Biochemistry and Biophysics, Polish Academy of Sciences, 02-106 Warsaw, Poland; Rega Institute of Medical Research, KU Leuven, B-3000 Leuven, Belgium; Institute of Pharmacology and Structural Biology, University of Toulouse, CNRS, 31400 Toulouse, France; Infrastructure Nationale de Protéomique, ProFI, UAR 2048 Toulouse, France; Institute of Biochemistry and Biophysics, Polish Academy of Sciences, 02-106 Warsaw, Poland; Clinical Research Centre, Medical University of Białystok, 15-089 Białystok, Poland; Life Sciences Center, Vilnius University, LT-10257 Vilnius, Lithuania; Faculty of Life Sciences, University of Bradford, BD7 1DP Bradford, United Kingdom

## Abstract

**Summary:**

Hydrogen–Deuterium Exchange Mass Spectrometry (HDX-MS) monitors deuterium uptake at the peptide level, in a time-dependent manner. It produces complex, multi-dimensional data that must be interpreted at minimum both the temporal and sequence levels. Specialized tools are therefore essential to preprocess, integrate, and analyze HDX-MS data and translate it into meaningful biological insights. HaDeX2 provides statistical inferences and their visualizations across five dimensions of HDX-MS data: protein sequence, time, biological states, peptide charge and experimental replicates.

**Availability and implementation:**

HaDeX2 is freely available as an R package (https://github.com/hadexversum/HaDeX2; https://doi.org/10.5281/zenodo.18543703) and web server (https://hadex2.mslab-ibb.pl/). To run the GUI locally, users should install a dedicated companion package (https://github.com/hadexversum/HaDeXGUI).

## 1 Introduction

Hydrogen–Deuterium Exchange Mass Spectrometry (HDX-MS) monitors the exchange of protein backbone amide hydrogen with deuterium during incubation in a D_2_O-based buffer. Following labeling under physiological conditions, the exchange is quenched in acidic conditions and low temperature to minimize deuterium-hydrogen back-exchange. Proteins are then digested using a proteolytic enzyme, before LC–MS analysis (Englander 2006). The measured deuterium uptake depends on the local stability and degree of hydrogen bonding, where rigid regions show less deuterium uptake while flexible or unstructured parts take up deuterium faster. Therefore, these changes in deuterium exchange provide direct insight into local dynamic and structural properties of the protein, information which HDX-MS is uniquely positioned to obtain.

While HDX-MS is conceptually simple, its analysis is complex due to the dimensionality of the resulting data, which spans five different dimensions: protein sequence, time, charge, biological state and replicate. The deuterium uptake can be measured only *in toto* on a peptide basis, which remains a key limitation of the widely used ’bottom-up’ experimental setup. Kinetics of deuterium exchange are monitored in a time dependent manner (5–6 time points), usually in three technical replicates (Masson et al. 2019).

The charge distribution of peptides varies across protein sequence, time points, conditions, and replicates. This variability can significantly affect results, as different charge states may show distinct uptake trajectories (Guttman et al. 2016). The complexity of HDX-MS data increases in comparative studies, where researchers assess how biological states (e.g. ligand binding) affect deuterium uptake trajectories. Such studies require specialized statistical methods to distinguish true HDX changes from biological or technical variability.

To address the complexity of HDX-MS data, we developed HaDeX2, a re-engineered version of the original HaDeX R package (Puchała et al. 2020). HaDeX2 enables both single-dimensional and integrated multi-dimensional analyses, with improved performance and scalability for large, complex protein systems, which are increasingly common in HDX-MS research (Sheff et al. 2017, Lesne et al. 2020).

## 2 Methods

Building on the multi-dimensional nature of HDX-MS data, HaDeX2 provides dedicated tools for exploring each facet individually or in combination. Users can examine deuterium uptake as a function of measurement time, sequence position, charge, biological state or replicate and integrate these observations into interpretable summaries, with a fully rewritten HaDeX2 backend ensuring that this multidimensional exploration is faster than in the previous iteration of our software (Supplementary Information 2.4).

Some of these summaries are well-standardized visual representations (described in depth in Supplementary Information 6.1–6.16), including butterfly plots, chicklet plots, and Woods plots, each of which enables time-dependent comparison of uptake dynamics across experimental conditions (Woods and Hamuro 2001, Pirrone et al. 2015). Given that these visualizations reveal only specific aspects of HDX-MS datasets, HaDeX2 incorporates further methods to enable deeper and more nuanced data exploration.

**Figure 1:**
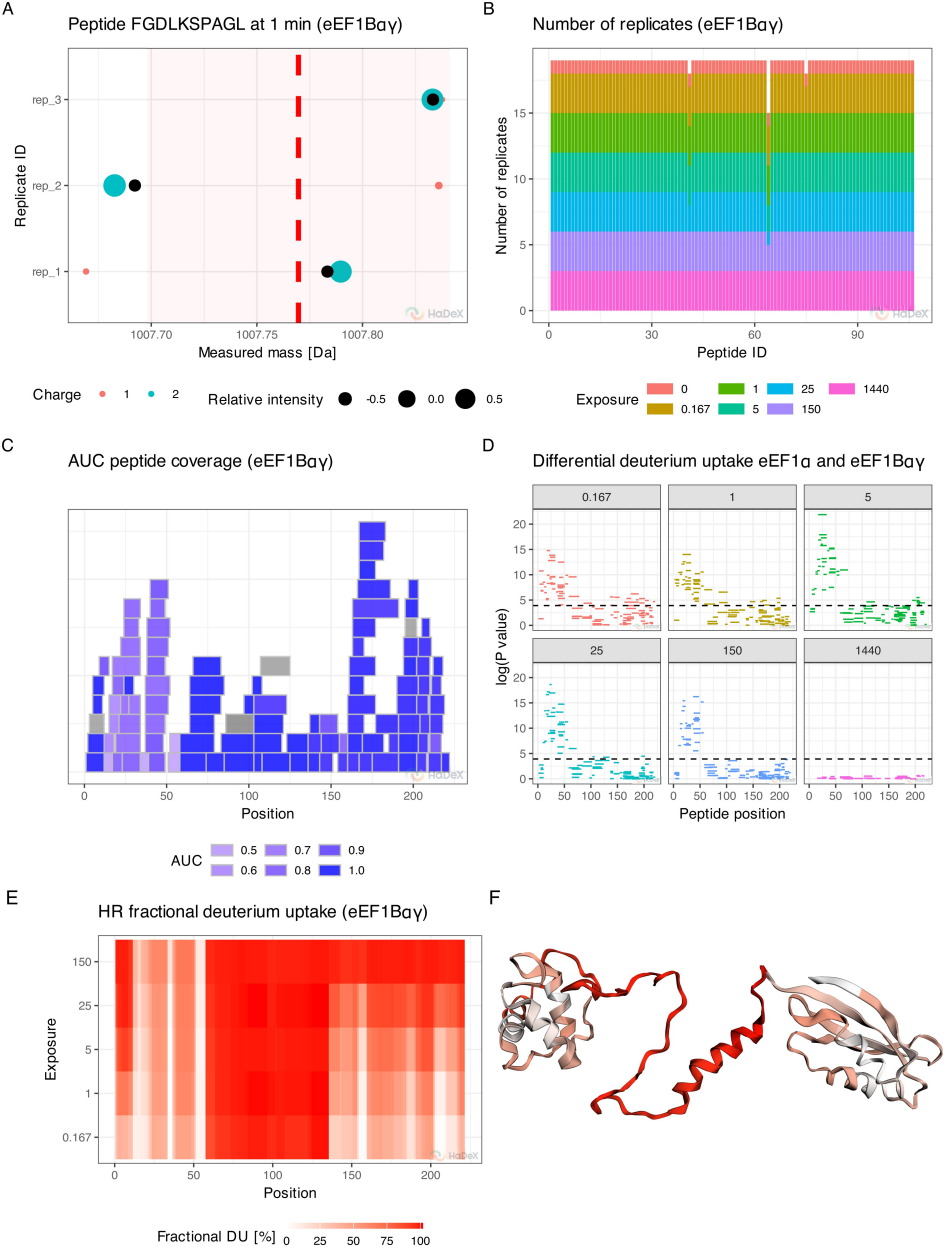
Example of novel functionalities of HaDeX2 using eEF1B*αγ* and eEF1B*α* subunits of the human guanine-nucleotide exchange factor (GEF) complex (PXD031783) (Bondarchuk et al. 2022). (A) The measurement variability plot shows the centroid values of isotopic envelopes for each possible charge state (represented by the point color) of a peptide across experimental replicates. Relative intensity (size of the points) is the weight of the contribution of a specific measurement to the total measured intensity for each replicate. The uncertainty interval (represented by the red area) represents the standard deviation of all measurements aggregated within each replicate (Supplementary information 5.3). (B) The replicate consistency plot displays the number of accepted mass measurements for peptides across the time course. (C) The coverage heatmap shows AUC values (area under the uptake curve) for peptides, alongside their length and position in the protein sequence. Greyed-out regions indicate peptides with insufficient data for AUC calculation. (D) The Manhattan plot displays the *P*-values of deuterium uptake differences between peptides in two biological states, shown separately for each time point. (E) The high-resolution heatmap, computed with ‘weighted averaging’ strategy (Keppel and Weis 2015), shows aggregated deuterium uptake in a single state. (F) The high-resolution deuterium uptake mapped onto the 3D protein structure at the 0.167 min time point sharing the color legend with the (E).

One of such unique methods is a measurement variability analysis, which describes deuterium uptake as a function of peptide position, replicate and charge (Fig. 1A, Supplementary Information 5.3). It highlights deviations that fall outside propagated uncertainty intervals, enabling rapid outlier detection and enhancing the transparency of quality control procedures.

Given the experimental variability inherent to HDX-MS, the number of biological and technical replicates may vary across time points and peptides. The replicate consistency plot (Fig. 1B) summarizes the distribution of replicates across the experimental matrix, enabling users to identify data imbalances or missingness which could harm subsequent statistical analysis.

The coverage map shows the location and size of proteolytic peptides across the whole protein sequence, providing an overview of peptide coverage, redundancy and local resolution (Supplementary Information 6.15). The map highlights regions with higher redundancy as more reliable for downstream interpretation. In addition, we extend this information through a coverage heatmap, which encodes additional peptide-level features such as the area under the deuterium uptake curve (AUC) (Fig. 1C) or the extent of back-exchange. These annotations facilitate the prioritization of regions for structural mapping and comparative analysis.

Following recent community recommendations, HaDeX2 incorporates a hybrid framework for significance testing in differential HDX analysis (Hageman and Weis 2019), along with dedicated error propagation methods (Weis 2021). These features ensure both statistical robustness and interpretability of the analysis, with final results presented visually in Manhattan (Fig. 1D) and volcano plots (Supplementary information 6.8 and 6.11).

A crucial strength of HaDeX2 lies in its interactive and reproducible web server. All plots support contextual customization and informative tooltips. Each visualization is directly linked to its underlying data, increasing the FAIRness of the data exploration. Users can export high-quality figures in SVG format with adjustable dimensions suitable for journal submission, or generate complete analysis reports for documentation purposes.

As bottom-up HDX-MS provides information restricted to the peptide level, HaDeX2 addresses this limitation by implementing a ’weighted averaging’ strategy. By systematically exploiting overlaps between peptides, the method increases spatial resolution of deuterium uptake, in some cases extending it to the residue level (Keppel and Weis 2015). This output can then be visualized as a heatmap (Fig. 1E) or directly visualized on protein structures provided in .mmcif or .pdb formats (Fig. 1F) using an internal viewer based on 3Dmol.js (Rego and Koes 2015). Moreover, high-resolution data can be exported in a standardized format compatible with HDX-Viewer (Bouyssié et al. 2019).

## 3 Conclusion and availability

The intrinsic complexity of HDX-MS data often leads to a too narrow focus on some of its dimensions. Therefore, we decided to build a more unified analytical environment for systematic exploration across all relevant facets. While retaining standard features encompassed by tools like HDXBoxeR (Janowska et al. 2024) and Deuteros (Lau et al. 2021), HaDeX2 extends functionality to uncover deeper structural insights or perform more robust quality control.

To maximize accessibility and prioritize user experience, HaDeX2 is available both as an open-source R package for integration in analysis workflows and as a web-based interface for broader adoption by experimental scientists. Additionally, HaDeX2 is compatible with leading vendor-specific preprocessing tools, including DynamX (Waters) and HDExaminer (Thermo Scientific). Detailed documentation provides step-by-step import instructions and outlines the principles necessary to support additional formats. We also present a complete HaDeX2 workflow showcasing its functionalities (Supplementary Information 7.2). At the R package level, the system provides flexible import mechanisms, allowing experienced users to construct customized preprocessing pipelines and integrate HaDeX2 into automated workflows.

A core strength of HaDeX2 lies in its explicit treatment of the multidimensional structure of HDX-MS data. The platform provides dedicated diagnostic plots and error models that account for variability in replicate number, time point coverage, peptide- and charge-state-level features, and biological condition with all funtionalities listed in the Supplementary Information, section 2.1. By propagating uncertainty into all stages of the workflow, including comparative analysis, HaDeX2 enhances the statistical robustness and interpretability of differential HDX studies. When statistical assumptions are violated (e.g. due to insufficient replication), hypothesis testing is automatically suppressed, ensuring analytical transparency.

To maximize accessibility, HaDex2 offers a straightforward weighted averaging approach to residue-level results. This avoids the need for specialist training and careful parameter selection or evaluation of results which is often required when using more advanced high-resolution algorithms as PyHDX (Smit et al. 2021), HR-HDX (Gessner et al. 2017), ExPfact (Skinner et al. 2019) or HRaDeX (Puchała et al. 2025). Given the current lack of benchmarking studies that clearly identify best-performing methods, we chose to await community consensus before incorporating such functionality. In the same spirit, we rely on classical statistical tests for HDX-MS analysis (Houde et al. 2011) and do not implement more advanced solutions (Claesen et al. 2021, Crook et al. 2023).

Unlike tools that address isolated components of the HDX-MS pipeline, such as back-end statistical testing, front-end visualization, or 3D structure mapping, HaDeX2 supports integrated exploration across all experimental dimensions. Considering all these factors, HaDeX2 seems to fulfill all criteria related to post-processing of HDX-MS data: multimodal analysis, differential analysis and high resolution HDX-MS (Stofella et al. 2024). Importantly, it is the only freely accessible platform unifying these capabilities, thus providing an open alternative to commercial solutions.

HaDeX2 moves beyond simply compiling existing algorithms and visualizations by introducing novel methods investigating all dimensions of HDX-MS data. Its open-source design, high accessibility, and FAIR-compliant reproducibility establish it as a robust platform for both reproducible studies today and integration of future advances in HDX-MS analysis.

## Supplementary Material

btag128_Supplementary_Data

## Data Availability

All datasets are included as datasets in the HaDeX R package.
