# Supplementary material for: HaDeX2: multi-dimensional analysis of hydrogen–deuterium exchange mass spectrometry data
Source: Bioinformatics. 2026 Mar 16;42(3):btag128. doi: 10.1093/bioinformatics/btag128 (PMC13020901; doi:10.1093/bioinformatics/btag128)
Supplement: btag128_Supplementary_Data [file btag128_supplementary_data.pdf]

Supplementary information to HaDeX2: multi-dimensional analysis of Hydrogen–Deuterium Exchange Mass Spectrometry data

Weronika Puchała, Krystyna Grzesiak, Dominik Rafacz, Michał Kistowski, Jochem H. Smit
Julien Marcoux, Michał Dadlez, Michał Burdukiewicz

Contents

1 Preface **2**

2 Comparison between versions **3**

2.1 Comparison of visualization types 3

2.2 New web-server features 3

2.3 Functionality mapping between HaDeX and HaDeX2 3

2.4 Performance benchmarking of HaDeX and HaDeX2 5

2.5 HaDeX2 design 8

3 Calculations **8**

3.1 Initial processing of the data 8

3.2 Calculation of the deuterium uptake 10

3.3 Differential values 11

3.4 Deuterium uptake averaging 11

3.5 Back-exchange 12

3.6 AUC 12

4 Statistics **12**

4.1 Uncertainty propagation 12

4.2 Hybrid testing 13

5 Quality control **14**

5.1 Replicates 14

5.2 Uncertainty 15

5.3 Measurement variability plot 16

5.4 Back-exchange 18

6 **Data visualization** **18**
6.1 Visualizing HDX-MS data . . . 18
6.2 Comparison plot . . . 18
6.3 Woods plot . . . 19
6.4 Butterfly plot . . . 21
6.5 Butterfly differential plot . . . 22
6.6 Chiclet plot . . . 23
6.7 Chiclet differential plot . . . 24
6.8 Volcano plot . . . 25
6.9 Uptake curve . . . 26
6.10 Uncertainty plot . . . 27
6.11 Manhattan plot . . . 28
6.12 High-resolution plot . . . 29
6.13 Differential High-resolution plot . . . 30
6.14 High-resolution on 3D structure . . . 31
6.15 Coverage heatmap . . . 32
6.16 Summary of the uptake plots . . . 34

7 **Example HDX-MS data analysis** **35**
7.1 Introduction . . . 35
7.2 Data analysis with HaDeX2 . . . 35

1 Preface

This document provides a comprehensive supplementary description of HaDeX2, an updated R package and web server for the analysis of hydrogen–deuterium exchange mass spectrometry (HDX-MS) data. It complements the main manuscript by providing the details on improvements over HaDeX 1.0, data processing and visualisation.

The opening section, *Comparison between versions*, contrasts HaDeX2 with the original HaDeX in terms of available functions and the speed of their execution. We follow it followed by *Calculations*, where we describe data preprocessing, replicate aggregation, mass calculation, and multiple formulations of deuterium uptake, including absolute, fractional, theoretical, and differential measures. Importantly, this section describes secondary descriptors as residue-level aggregation and area under the uptake curve. The subsequent *Statistics* section is devoted to statistical inference on HDX-MS data as uncertainty propagation rules or hybrid testing employed by HaDeX 2.0.

Data visualization describes all plot types available in HaDeX2, clarifying the purpose, strengths and limitations. It also introduces graphical representations of results defined in previous sections, linking computational and statistical outputs to interpretable visual summaries. These visualisations include AUC- or back-exchange-based coverage heatmaps and Manhattan or volcano plots summarizing the outcomes of the hybrid testing procedure. The final section, *Example HDX-MS data analysis*, presents an end-to-end case study that demonstrates the capabilities of HaDeX2 web server.

2 Comparison between versions

Due to the wider scope of the second version of HaDeX, we developed it as an independent package from the original iteration (Puchała et al. 2020). This section outlines the differences between HaDeX 2.0 and HaDeX 1.0, highlighting the extended capabilities and improved computational performance.

2.1 Comparison of visualization types

We first compare the visualization methods implemented in the package and web-server versions.

plot_type	HaDeX	HaDeX2
comparison	TRUE	TRUE
woods	TRUE	TRUE
uptake curve	TRUE	TRUE
diff uptake curve	FALSE	TRUE
butterfly	FALSE	TRUE
diff butterfly	FALSE	TRUE
chiclet	FALSE	TRUE
diff chiclet	FALSE	TRUE
heatmap	FALSE	TRUE
diff heatmap	FALSE	TRUE
3D structure	FALSE	TRUE
volcano	FALSE	TRUE
manhattan	FALSE	TRUE
uncertainty	FALSE	TRUE
coverage	TRUE	TRUE
coverage heatmap	FALSE	TRUE
measurement variablity	FALSE	TRUE
mass uptake curve	FALSE	TRUE

2.2 New web-server features

One of the most fundamental changes was the extensions of interactivity and reproducibility of web servers.

option	HaDeX	HaDeX2
tooltips	TRUE	TRUE
helpers	TRUE	TRUE
tabular data	TRUE	TRUE
times next to each other	FALSE	TRUE
export to external tools	FALSE	TRUE

In the table above, *Tabular data* indicates whether the values underlying a given visualization are available to download in a tabular form. *Times next to each other* refers to the option of displaying measurements from multiple time points either within a single plot or as a series of adjacent plots, each representing an individual time point. *Export to external tools* means an option to export processed data to external applications such as HDXViewer or ChimeraX.

2.3 Functionality mapping between HaDeX and HaDeX2

The table below compare the functions implemented in HaDeX2 with their counterparts in HaDeX.

HaDeX	HaDeX2

HaDeX_gui	HaDeX_GUI
add_stat_dependency	add_stat_dependency
calculate_confidence_limit_values	calculate_confidence_limit_values
calculate_kinetics	calculate_kinetics
calculate_state_deuteration	create_state_uptake_dataset
comparison_plot	plot_state_comparison
plot_coverage	plot_coverage
plot_kinetics	plot_uptake_curve
plot_position_frequency	plot_overlap_distribution
read_hdx	read_hdx
reconstruct_sequence	reconstruct_sequence
woods_plot	plot_differential
NA	HaDeXify
NA	calculate_MHP
NA	calculate_aggregated_diff_uptake
NA	calculate_aggregated_test_results
NA	calculate_aggregated_uptake
NA	calculate_auc
NA	calculate_back_exchange
NA	calculate_diff_uptake
NA	calculate_exp_masses
NA	calculate_exp_masses_per_replicate
NA	calculate_p_value
NA	calculate_peptide_kinetics
NA	calculate_state_uptake
NA	create_aggregated_diff_uptake_dataset
NA	create_aggregated_uptake_dataset
NA	create_control_dataset
NA	create_diff_uptake_dataset
NA	create_kinetic_dataset
NA	create_overlap_distribution_dataset
NA	create_p_diff_uptake_dataset
NA	create_p_diff_uptake_dataset_with_confidence
NA	create_replicate_dataset
NA	create_state_comparison_dataset
NA	create_uptake_dataset
NA	get_n_replicates
NA	get_peptide_sequence
NA	get_protein_coverage
NA	get_protein_redundancy
NA	get_replicate_list_sd
NA	get_residue_positions
NA	get_structure_color
NA	install_GUI
NA	plot_aggregated_differential_uptake
NA	plot_aggregated_uptake
NA	plot_aggregated_uptake_structure
NA	plot_amino_distribution
NA	plot_butterfly
NA	plot_chiclet
NA	plot_coverage_heatmap

NA	plot_differential_butterfly
NA	plot_differential_chiclet
NA	plot_differential_uptake_curve
NA	plot_manhattan
NA	plot_overlap
NA	plot_peptide_charge_measurement
NA	plot_peptide_mass_measurement
NA	plot_position_frequency
NA	plot_quality_control
NA	plot_replicate_histogram
NA	plot_replicate_mass_uptake
NA	plot_uncertainty
NA	plot_volcano
NA	prepare_hdxviewer_export
NA	quality_control_dataset
NA	show_aggregated_uptake_data
NA	show_coverage_heatmap_data
NA	show_diff_uptake_data
NA	show_diff_uptake_data_confidence
NA	show_overlap_data
NA	show_p_diff_uptake_data
NA	show_peptide_charge_measurement
NA	show_peptide_mass_measurement
NA	show_quality_control_data
NA	show_replicate_histogram_data
NA	show_summary_data
NA	show_uc_data
NA	show_uptake_data
NA	update_hdexaminer_file

2.4 Performance benchmarking of HaDeX and HaDeX2

For each pair of functions in the previous section, we can assess relative execution speed using the exemplary dataset as a controlled reference for comparison. We concentrate on six major tasks: reading data file, plotting (and preparing data) uptake curve for a single peptide, comparison plot of two biological states, differential Woods plot with difference between two states, reconstruction of the protein sequence and computation of confidence limits.

We performed the benchmark utilizing the code shown below.

```
library(HaDeX)

dat_HaDeX <- HaDeX::read_hdx(system.file(package = "HaDeX2", "HaDeX/data/alpha.csv"))
dat_HaDeX2 <- HaDeX2::read_hdx(system.file(package = "HaDeX2", "HaDeX/data/alpha.csv"))

version_benchmark <- microbenchmark(
  list = alist(`HaDeX_1. Read input` = HaDeX::read_hdx(system.file(package = "HaDeX2",
                                                                   "HaDeX/data/alpha.csv")),
               `HaDeX2_1. Read input` = HaDeX2::read_hdx(system.file(package = "HaDeX2",
                                                                     "HaDeX/data/alpha.csv")),
               `HaDeX_2. Plot uptake curve` = {
                 HaDeX::calculate_kinetics(dat = dat_HaDeX,
```

```
                             sequence = "GFGDLKSPAGL",
                             state = "Alpha_KSCN",
                             start = 1, end = 11,
                             time_in = 0, time_out = 1440) %>%
    HaDeX::plot_kinetics(kin_dat = .)},
`HaDeX2_2. Plot uptake curve` = {
  HaDeX2::calculate_peptide_kinetics(dat = dat_HaDeX2,
                                     sequence = "GFGDLKSPAGL",
                                     state = "Alpha_KSCN",
                                     start = 1, end = 11,
                                     time_0 = 0, time_100 = 1440) %>%
    HaDeX2::plot_uptake_curve(uc_dat = .)},
`HaDeX_3. Plot comparison` = {
  HaDeX::prepare_dataset(dat = dat_HaDeX,
                         in_state_first = "Alpha_KSCN_0",
                         chosen_state_first = "Alpha_KSCN_1",
                         out_state_first = "Alpha_KSCN_1440",
                         in_state_second = "ALPHA_Gamma_0",
                         chosen_state_second = "ALPHA_Gamma_1",
                         out_state_second = "ALPHA_Gamma_1440") %>%
    HaDeX::comparison_plot(calc_dat = .,
                           theoretical = FALSE,
                           relative = TRUE,
                           state_first = "Alpha_KSCN",
                           state_second = "ALPHA_Gamma")},
`HaDeX2_3. Plot comparison` = {
  HaDeX2::create_state_comparison_dataset(dat = dat_HaDeX2,
                                          states = c("Alpha_KSCN",
                                                     "ALPHA_Gamma"),
                                          time_0 = 0, time_100 = 1440) %>%
    HaDeX2::plot_state_comparison(uptake_dat = .,
                                  theoretical = FALSE,
                                  fractional = TRUE,
                                  time_t = 1)},
`HaDeX_4. Plot Woods` = {
  HaDeX::prepare_dataset(dat = dat_HaDeX,
                         in_state_first = "Alpha_KSCN_0",
                         chosen_state_first = "Alpha_KSCN_1",
                         out_state_first = "Alpha_KSCN_1440",
                         in_state_second = "ALPHA_Gamma_0",
                         chosen_state_second = "ALPHA_Gamma_1",
                         out_state_second = "ALPHA_Gamma_1440") %>%
    HaDeX::woods_plot(calc_dat = .,
                      theoretical = FALSE,
                      relative = TRUE,
                      confidence_limit = 0.98,
                      confidence_limit_2 = 0.98)},
`HaDeX2_4. Plot Woods` = {
  HaDeX2::calculate_diff_uptake(dat = dat_HaDeX2,
                                states = c("Alpha_KSCN", "ALPHA_Gamma"),
                                time_t = 1, time_0 = 0, time_100 = 1440) %>%
    HaDeX2::plot_differential(diff_uptake_dat = .,
                              time_t = 1,
```

Table 2: Median speed of function execution (in miliseconds).

task	HaDeX	HaDeX2	Runtime ratio
1. Read input	34.83070	28.86460	0.8287115
2. Plot uptake curve	171.86675	65.38155	0.3804200
3. Plot comparison	186.86305	59.34140	0.3175663
4. Plot Woods	201.53030	77.75905	0.3858430
5. Calculate confidence limit	172.91570	53.16090	0.3074383
6. Reconstruct sequence	24.18105	15.78765	0.6528935

```
                                   theoretical = FALSE,
                                   fractional = TRUE,
                                   show_houde_interval = TRUE,
                                   confidence_level = 0.98)},
               `HaDeX_5. Calculate confidence limit` = {
                 HaDeX::prepare_dataset(dat = dat_HaDeX,
                                        in_state_first = "Alpha_KSCN_0",
                                        chosen_state_first = "Alpha_KSCN_1",
                                        out_state_first = "Alpha_KSCN_1440",
                                        in_state_second = "ALPHA_Gamma_0",
                                        chosen_state_second = "ALPHA_Gamma_1",
                                        out_state_second = "ALPHA_Gamma_1440") %>%
                   HaDeX::calculate_confidence_limit_values(calc_dat = .,
                                                            confidence_limit = 0.98,
                                                            theoretical = FALSE,
                                                            relative = TRUE)},
                `HaDeX2_5. Calculate confidence limit` = {
                 HaDeX2::calculate_diff_uptake(dat = dat_HaDeX2,
                                               states = c("Alpha_KSCN", "ALPHA_Gamma"),
                                               time_0 = 0, time_100 = 1440, time_t = 1) %>%
                   HaDeX2::calculate_confidence_limit_values(diff_uptake_dat = .,
                                                             confidence_level  = 0.98,
                                                             theoretical = FALSE,
                                                             fractional = TRUE)},
               `HaDeX_6. Reconstruct sequence` = HaDeX::reconstruct_sequence(dat = dat_HaDeX),
               `HaDeX2_6. Reconstruct sequence` =  HaDeX2::reconstruct_sequence(dat = dat_HaDeX2)

               )
)
```

The *microbenchmark* package operates by repeatedly executing each command 100 times o obtain a stable and representative estimate of execution time.

Across all tasks, the reported values represent a runtime ratio (HaDeX2/HaDeX) consistently below one, indicating that HaDeX2 is faster than HaDeX for every measured operation. The strongest speedups, corresponding to the lowest ratios, are observed for plotting functions, calculating confidence limits, and plotting uptake curves, whereas input reading and sequence reconstruction show comparatively smaller, though still meaningful, reductions in execution time. In the case of input reading, the modest speed-up results from the fact that this functionality has substantially expanded in-built quality control in HaDeX2, where additional validation steps intentionally constrain maximal speed in favor of improved data integrity.

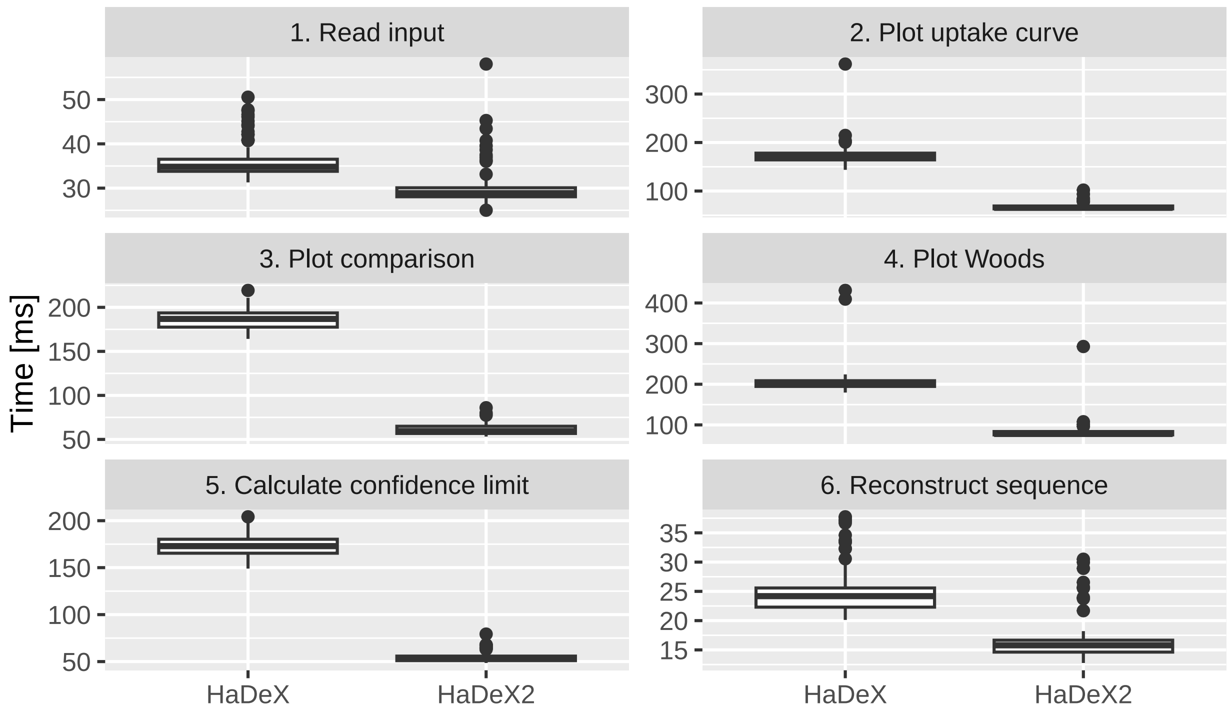

Figure 1: Benchmark results.

2.5 HaDeX2 design

The first version of HaDeX was developed quickly to address immediate data analysis challenges. As knowledge in the field expanded, it became necessary to extend the package's functionality. This required a carefully planned redesign. The package is now built from small, modular computing blocks—encapsulated functions that each perform a single task. Datasets are created by combining these functions. This design allows individual components to be tested independently and improves code readability through self-explanatory function names (calculate_ provides results for specific time point, but create_dataset_ for all time points). The parameter naming conventions were also simplified. In addition, the graphical user interface was rewritten from scratch using Shiny modules to ensure clear separation and encapsulation of features.

3 Calculations

This section describes how HaDeX2 processes raw HDX-MS data files into aggregated datasets for downstream analysis and visualization. It details the data processing steps, the computational workflow used to derive analytical quantities and the resulting outputs. Visualization methods for the derived values are described in the section 6.1.

3.1 Initial processing of the data

Let's start with a glimpse of the data file - in this case, in `cluster` format from DynamX.

```r
library(HaDeX2)
head(alpha_dat, 6)
#>       Protein Start   End     Sequence Modification MaxUptake      MHP       State Exposure
#>        <char> <int> <int>       <char>        <lgcl>     <int>    <num>      <char>    <num>
#> 1: db_eEF1Ba     1    11 GFGDLKSPAGL           NA         9 1061.563 ALPHA_Gamma    0.000 Tania_1611
#> 2: db_eEF1Ba     1    11 GFGDLKSPAGL           NA         9 1061.563 ALPHA_Gamma    0.000 Tania_1611
#> 3: db_eEF1Ba     1    11 GFGDLKSPAGL           NA         9 1061.563 ALPHA_Gamma    0.167  Tania_161
#> 4: db_eEF1Ba     1    11 GFGDLKSPAGL           NA         9 1061.563 ALPHA_Gamma    0.167  Tania_161
#> 5: db_eEF1Ba     1    11 GFGDLKSPAGL           NA         9 1061.563 ALPHA_Gamma    0.167  Tania_161
#> 6: db_eEF1Ba     1    11 GFGDLKSPAGL           NA         9 1061.563 ALPHA_Gamma    0.167  Tania_161
```

As we see, the data file has a very specific structure and is not informative yet. In the file, we have m/z values for each z value (charge) for each time point ($Exposure$) for each state of each peptide, repeated as many times as measurement was repeated (each measurement should be repeated at least three times).

Our aim is to have one result with an uncertainty of the measurement per each peptide in each biological state in each measured time point - data in this format allows further calculations, e.q. calculating deuterium uptake values.

Within each replicate of the measurement (we recognize each replicate by the $File$ value), the m/z values are provided for each possible z value. The m/z values are in the $Center$ column, as it is a geometrical centroid calculated from the isotopic envelope. Firstly, we have to calculate the mass value, measured experimentally:

$$expMass = z \times (Center - protonMass) \tag{1}$$

To aggregate data from different z values, we have to calculate the mean mass weighted by intensity.

$$aggMass = \frac{1}{N} \sum_{k=1}^{N} Inten_k \cdot pepMass_k \tag{2}$$

.

Where:

- $aggMass$ - mass of the peptide [Da], average from replicates,
- $Inten_k$ - intensity of the measurement,
- $pepMass$ - mass of the peptide, calculated using Equation 1.

As we use the aggregated result from the replicates, we need to calculate an uncertainty associated with the measurement. We use the mean value as the final result, so we need to calculate error as a standard deviation of the mean, according to the Equation 3:

$$u(\vec{x}) = \sqrt{\frac{\sum_{i=1}^{n} (x_i - \overline{x})^2}{n(n-1)}} \tag{3}$$

.

Where:

- x_i - measured value from a replicate,
- $\overline{x}$ - mean value from all of the replicates,
- n - number of replicates.

Now we have the format we want for further calculations.

3.2 Calculation of the deuterium uptake

HaDeX package provides the calculated values in different forms. All of them are provided with associated uncertainty of the measurement. All of the uncertainties are derived from the formula - the Law of propagation of uncertainty:

$$u_c(y) = \sqrt{\sum_k \left[\frac{\partial y}{\partial x_k} u(x_k)\right]^2}$$

3.2.1 Deuterium uptake

Deuterium uptake is the increase of the mass of the peptide in time t. The minimal exchange control m_{t_0} is mass measured directly after adding the buffer (before the start of the exchange), and m_t is the mass measured in chosen time point t. The value is in Daltons [Da].

$$D = m_t - m_{t_0}$$

The uncertainty associated with deuterium uptake [Da] (based on equation 3):

$$u_c(D) = \sqrt{u(m_t)^2 + u(m_{t_0})^2}$$

3.2.2 Fractional deuterium uptake

Fractional deuterium uptake is the ratio of the increase of the mass in time t to the maximal exchange control. The maximal exchange control $m_{t_{100}}$ is measured after a long time (chosen by the experimenter, usually 1440 min = 24 h). It is assumed that after this long time, the exchange is finished. The minimal exchange control m_{t_0} is mass measured directly after adding the buffer (before the start of the exchange), and m_t is the mass measured in chosen time point t. This value is a percentage value [%].

$$D_{frac} = \frac{m_t - m_{t_0}}{m_{t_{100}} - m_{t_0}}$$

The uncertainty associated with fractional deuterium uptake [%] (based on equation 3):

$$u_c(D_{frac}) = \sqrt{\left[\frac{1}{m_{t_{100}} - m_{t_0}} u(m_t)\right]^2 + \left[\frac{m_t - m_{t_{100}}}{(m_{t_{100}} - m_{t_0})^2} u(m_{t_0})\right]^2 + \left[\frac{m_{t_0} - m_t}{(m_{t_{100}} - m_{t_0})^2} u(m_{t_{100}})\right]^2}$$

3.2.3 Theoretical deuterium uptake

Theoretical deuterium uptake is the increase of mass in time t compared with the theoretical value of the peptide mass without any exchange (MHP - a mass of the singly charged monoisotopic molecular ion), and m_t is the mass measured in chosen time point t. This value is in daltons [Da]:

$$D_{theo} = m_t - MHP$$

The uncertainty associated with theoretical deuterium uptake [Da] (the MHP value is a constant without measurement uncertainty - based on the equation 3):

$$u(D_{theo}) = u(m_t)$$

3.2.4 Theoretical fractional deuterium uptake

Theoretical fractional deuterium uptake is the ratio of the increase of mass in time t compared with a theoretical value of the mass of the peptide without any exchange to the possible theoretical increase of the mass, based on the maximal potential uptake of the peptide (based on the peptide sequence). This value is a percentage value [%].

$$D_{theo,frac} = \frac{m_t - MHP}{MaxUptake \times protonMass}$$

The uncertainty associated with theoretical fractional deuterium uptake [%] (based on the equation 3):

$$u(D_{theo,frac}) = \left| \frac{1}{MaxUptake \times protonMass} u(D_t) \right|$$

3.3 Differential values

Differential value is the way to see how the deuterium uptake differs between two biological states. It allows seeing if the possible difference is statistically important (more information below). This value is calculated as the difference between the previously described (in a chosen form) deuterium uptake of the first and second states.

$$diff = D_1 - D_2$$

The uncertainty associated with the difference of deuterium uptake (based on the equation 3):

$$u_c(diff) = \sqrt{u(D_1)^2 + u(D_2)^2}$$

The convenient way to present results calculated as described is the comparison plot and differential plot (Woods' plot).

3.4 Deuterium uptake averaging

Averaging of the deuterium uptake values is inspired by an already published solution (Keppel and Weis 2015). This process can be conducted of any deuterium uptake variation - fractional or absolute, with theoretical or experimental control, single-state or differential.

For each residue i there is a subset of peptides (n) covering said residue. Then, the final $D_{i,agg}$ is calculated from the subset of D_k (where $0 < k < n$), with weights w_k inverse proportional to the max uptake of peptide ($MaxUptake_k$) - the shortest the peptide the highest the possibility that the uptake took place in said residue:

$$w_k = \frac{\frac{1}{MaxUptake_k}}{\sum_n \frac{1}{MaxUptake_k}}$$

The weights are normalized:

$$\sum_n w_k = 1$$

Then, the $D_{i,agg}$ is a weighted average of set of D_n.

$$D_{i,agg} = \frac{\sum_n w_k \cdot D_k}{\sum_n w_k}$$

This process is done for each residue i to cover whole protein data.

As for previous values, also for $D_{i,agg}$ the uncertainty is calculated:

$$u(D_{i,agg}) = \sqrt{\sum_n \left[\frac{w_k \cdot U(D_k)}{\sum_n w_k} \right]^2}$$

3.5 Back-exchange

Back-exchange is a reverse exchange - from already exchange deuterium back to hydrogen.

Back-exchange (bex) is calculated for each peptide and is defined as the difference between fractional theoretical deuterium uptake of the experimental maximal deuterium control and 100% of exchange.

$$bex = 100 - D_{theo,frac}(t_{100})$$

3.6 AUC

The Area Under the Curve (AUC) represents the integral of the deuterium uptake curve. In this context, higher AUC values characterize rapidly exchanging peptides, while lower values signify minimal exchange. To constrain the results to a $[0, 1]$ range, the AUC is normalized against the maximum theoretical uptake at the last measured time point. Consequently, an AUC exceeding 1 serves as a diagnostic indicator of back-exchange.

$$\text{AUC} = \sum_i^{n-1} (D_{frac,i+1} + D_{frac,i}) \cdot (t_{\text{norm},i+1} - t_{\text{norm},i}) \cdot \frac{1}{2}$$

4 Statistics

Here, we sum up the statistics in HaDeX2. Some of the elements are discussed in other articles in appropriate places, but this article gathers this information in one place.

4.1 Uncertainty propagation

The propagation of uncertainty (Puchała et al. 2020; Weis 2021) is necessary when we are transforming the measured values. In HDX-MS, we repeat the measurements in triplicate in order to calculate the uncertainty of mass measurement. However, when transforming mass measurements into deuterium uptake, we need to propagate mass measurement uncertainty, using the Law of Propagation of Uncertainty (Joint Committee for Guides in Metrology 2008):

$$u_c(y) = \sqrt{\sum_k \left[\frac{\partial y}{\partial x_k} u(x_k) \right]^2}$$

Where:

- $u_c(y)$ - combined uncertainty of value y, where y is a function of x_k,
- x_k - values for which the uncertainty is known.

This is a generic equation used for the propagation of uncertainty in functions of multiple variables. It is instantiated for deuterium uptake in its appropriate formulations, as the specific expressions differ depending on the parameters of the calculation, and is described in detail in the dedicated subsection 3.2.

4.2 Hybrid testing

HaDeX offers the hybrid testing procedure (Hageman and Weis 2019) because it addressed the trade-off between false positives and loss of power in the analysis of HDX-MS data. The hybrid test applies a two-stage decision rule to identify significantly different deuterium uptakes. First, the observed difference in deuterium uptake must exceed a globally estimated uncertainty threshold derived from pooled experimental standard deviations, ensuring that the effect is larger than expected measurement error. Only differences passing this magnitude-based filter are then evaluated using a replicate-aware hypothesis test, implemented as a Welch's t-test, to assess whether the observed difference is statistically supported given the individual variances. A difference is considered significant only when both criteria are satisfied simultaneously.

Although the hybrid test offers superior statistical power compared to more classical statistical approaches, it is more data-intensive and therefore can be performed only when the user provides the experiment with at least three experimental replicates.

4.2.1 Houde interval

This test is done for the time points chosen for a given plot e.q. for the volcano plot, where presenting multiple time points of measurement, we take the values from all of the presented time points. However, for Woods Plot we only take into account only one time point - presented on the plot.

Houde interval (Houde, Berkowitz, and Engen 2011) is calculated based on the uncertainty of the measurement - or, more precisely, the propagated uncertainty of the deuterium uptake (in the same form as values presented on the plot). As described in the equation:

$$interval = \frac{\sum_i^n u_c(du_n)}{i} * tvalue(k)$$

where:

- n - number of peptides,
- $u_c(du_n)$ - uncertainty of the deuterium uptake for ith peptide,
- $tvalue$ - value for test for k replicates from the table,
- k - number of the replicates of the experiment.

$tvalue$ is calculated as follows, using R-function `qt`:

$$tvalue = qt(c(alpha/2, 1 - alpha/2), df = k - 1)$$

where the degree of freedom is the number of replicates minus one, and alpha is $1 -$ confidence limit for the desired confidence level (usually 0.98).

Basically, we take the mean uncertainty of deuterium uptake and widen this range by the appropriate value to get an interval. Values under the interval are too small and may be mistaken with the uncertainty. We are not interested in them.

4.2.2 Student's t-test

In order to use student t-test, we need at least three values from each group - in the case of the differential analysis - at least three replicate values at given time for each biological state.

This test shows us if the values are from two different distributions (desired option) or from one - and are the same. We are not interested in the latter case.

We use the unpaired Student's t-test to calculate p-value. The null hypothesis is that this two distributions are the same. If calculated p-value exceeded limit set for chosen confidence limit, we reject the null hypothesis and assume that the distributions are different.

To calculate p-value we use base R-function `t.test`

```
t.test(x = st_1, y = st_2,
       paired = FALSE,
       alternative = "two.sided",
       conf.level = confidence_level)$p.value
```

where st_1 is a set of values from the first state, and st_2 from the second.

If this option is chosen, we adjust the p-value using appropriate adjustment method (with three options: 'none', 'BH' and 'bonferroni'):

```
p.adjust(p_dat[["P_value"]], method = p_adjustment_method)
```

p-value is usually presented in the form of $-log$(p-value), e.g. on the volcano plot.

5 Quality control

```
library(HaDeX2)
```

Data analysis is vital step, but firstly, we must make sure that the experiment was conducted correctly. How to do that using HaDeX functionalities? There are multiple ways to do that. Unfortunately, our example data is good quality, but we will discuss possible indicators of data that needs to be re-checked.

5.1 Replicates

Firstly, we check the number of replicates. Although the experiment is conducted in known number of times, sometimes during the manual curation of isotopic envelopes, some of the spectra are disqualified. Here, we check the remaining number of replicates of the already curated dataset.

It is advised to have at least three replicates of the experiment. Lesser number limits the statistical analysis - e.q. for calculating P-value using T-student test minimum of three replicates are needed. If there are only two replicates, the uncertainty can be calculated.

In the example plot below, we see that there are three peptides in this state that are lacking values. One peptide - with considerably lack of replicates potentially should be eliminated from the peptide pool. Majority of peptides have a stable number of replicates - in this case three, with exception of no-deuterated control sample, measured only once.

```
rep_dat <- create_replicate_dataset(alpha_dat)
plot_replicate_histogram(rep_dat)
```

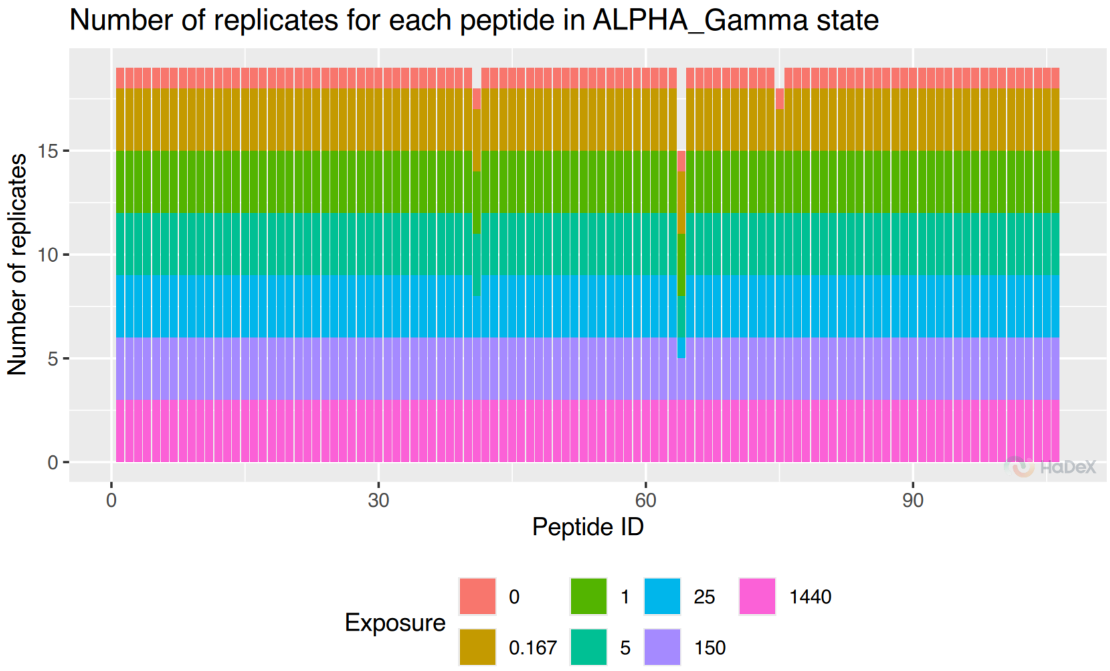

5.2 Uncertainty

Uncertainty plot presents the uncertainty of the measurement for one state at the time. In this case, we present the time points separately. There is no uncertainty calculated for time point 0 min, as it is treated as undeuterated control and measured only once. For other time points, as the measurement was conducted in triplicate, there are values for measured mass. As the measured mass is in daltons, also the uncertainty is presented in daltons. Thus, we can establish a threshold of 1 dalton - indicating one exchange between proton and deuter. Any uncertainty exceeding - or coming close - to this threshold should be carefully checked.

For correctly conducted experiment, the uncertainty for all peptides in all time points should be close to 0.

In the example below, all measurement have acceptable level of uncertainty. Of course, those values differ depending on the region or peptide length, but overall are not exceeding 0.25 Da.

```
plot_uncertainty(alpha_dat, state = "Alpha_KSCN")
```

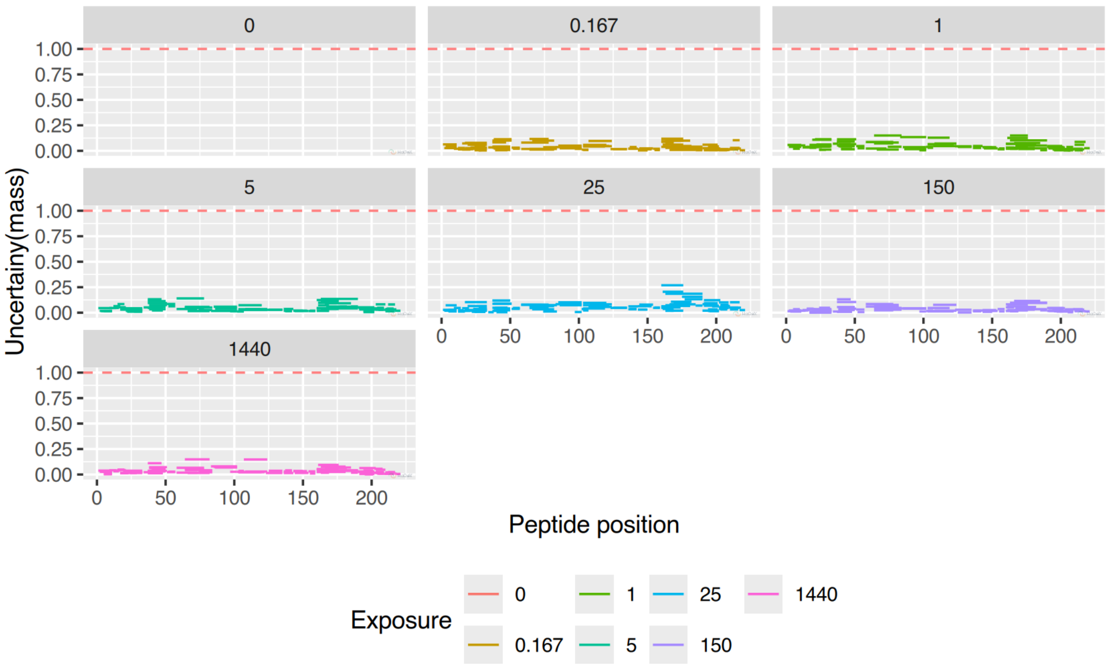

5.3 Measurement variability plot

Once we have the overview of the situation, we can dig deeper into the peptides of interest.

Below, we present the measurement variability plot for peptide "GDGDLKSPAGL" in one state, for three replicates.The process of two-step aggregation is described in the article `transformation`, but this plot allows investigation of each measurement.

Here, in case of peptide "GDGDLKSPAGL", there were only two possible charge values: 1 and 2. The mass was measured for each of them separately, and the centroid value from isotopic envelope was calculated, as well as the appropriate intensity. On the Y axis of the plot, there are replicates: and each replicate has its two points for each charge - with colors indicating charge and size indicating intensity. Then, those two values within replicate are aggregated - as a weighted mean, with intensity being the weight. For each replicate we have now one value - black dot. Those values are averaged with accompanied standard deviation - our final aggregated mass measurement from all the replicates. The final value is shown as the horizontal dotted line, with its sd value indicated by the red area.

```
plot_peptide_mass_measurement(alpha_dat)
```

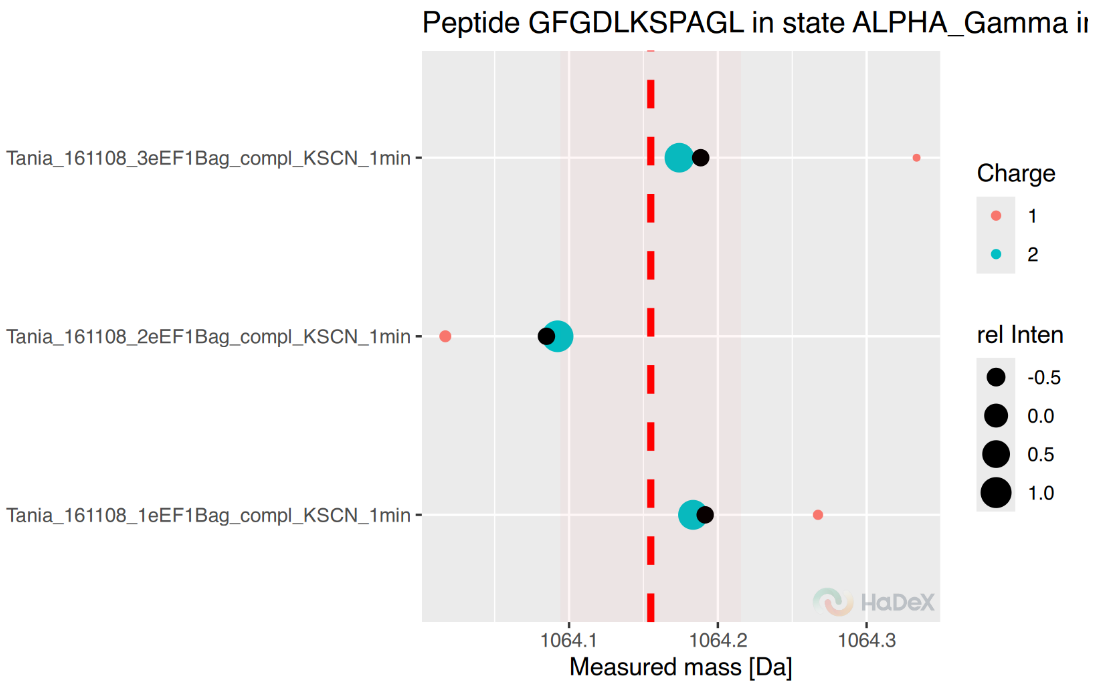

The peptide recognized as under-measured using previous plot, is peptide “CVRSIQA” in state eEF1Bγ. We analyze the mass uptake with regards to replicates (aggregated charge) and we see that for time point 25 min only one measurement was accepted, and for other time points two, thus blocking the possibility of calculating statistical significance when using this peptide in comparative analysis.

```r
plot_replicate_mass_uptake(alpha_dat, sequence = "CVRSIQA", aggregated = TRUE)
```

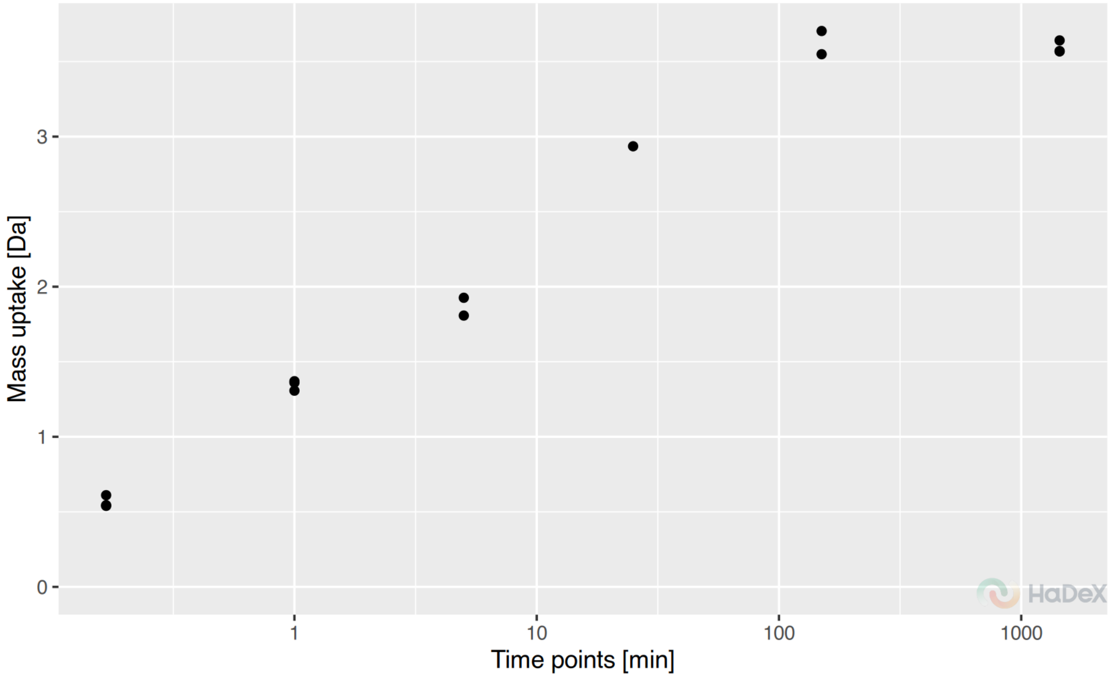

5.4 Back-exchange

Important measure of quality of experiment is back-exchange. Usually, expected value of back-exchange should be around 30%, possibly higher for shorter peptides.

```
bex_dat <- calculate_back_exchange(alpha_dat, state = "Alpha_KSCN")
plot_coverage_heatmap(bex_dat, value = "back_exchange")
```

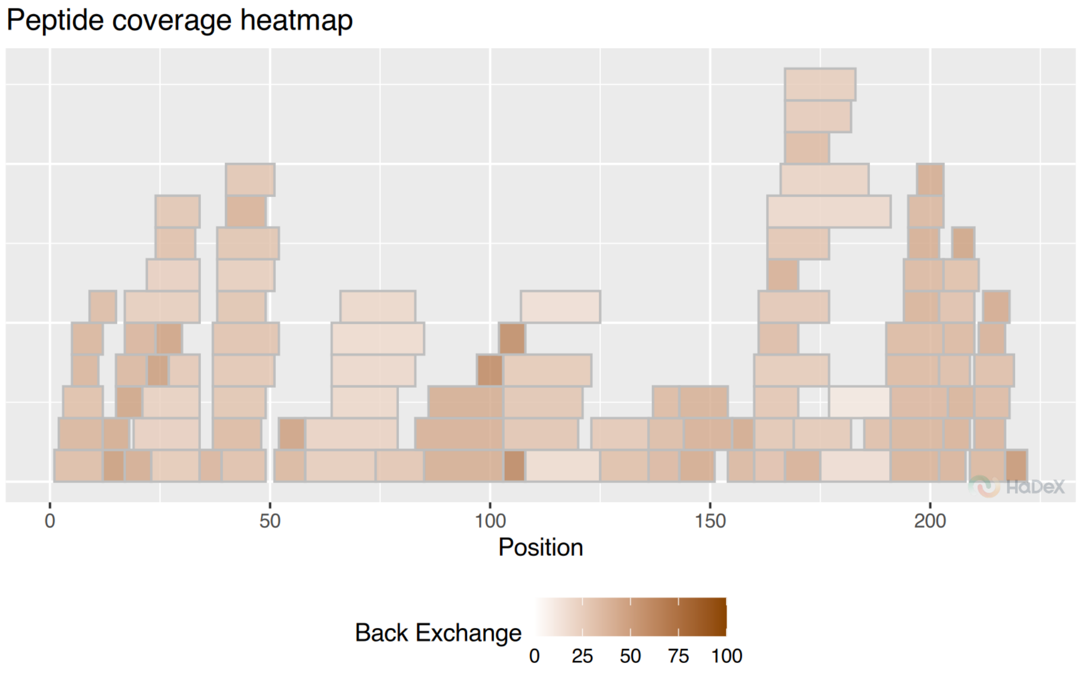

6 Data visualization

6.1 Visualizing HDX-MS data

Data visualization is a crucial part of the experimental data analysis. The forms of visualization should be adjusted to highlight the essential result and tailored to satisfy personal needs.

This article describes the visualization methods available in HaDeX2 and explains how different plot types can be used to interpret HDX-MS data. It focuses on the biological questions each visualization addresses rather than implementation details.

The analyzed protein is the eEF1Bα subunit of the human guanine-nucleotide exchange factor (GEF) complex (eEF1B), measured in Mass Spectrometry Lab in Institute of Biochemistry and Biophysics Polish Academy of Sciences (Bondarchuk et al. 2022). In the one-state classification, we will focus on pure alpha state - eEF1Bα. The comparative analysis is conducted between eEF1Bα and eEF1Bα in presence of eEF1Bγ.

6.2 Comparison plot

The comparison plot presents deuterium uptake of the peptides in a given time point, with information on the length of the peptide and their position in the protein sequence. It allows comparison of the results of different biological states.

Example In the comparison plot below, we see the fractional deuterium uptake for all three possible states: eEF1Bα, eEF1Bα in presence of eEF1Bγ and in presence of eEF1Bβ, from protein eEF1B. The values are calculated for the time point 1 min. The length of the segments represents the length of the peptide and the position in the protein sequence. The error bars indicate the uncertainty of the measurement.

```
create_state_comparison_dataset(alpha_dat, time_t = 1) %>%
  plot_state_comparison(., fractional = TRUE) +
  labs(x = "Position in sequence",
       y = "Fractional deuterium uptake [%]",
       title = "Measurement after 1 min of exchange")
```

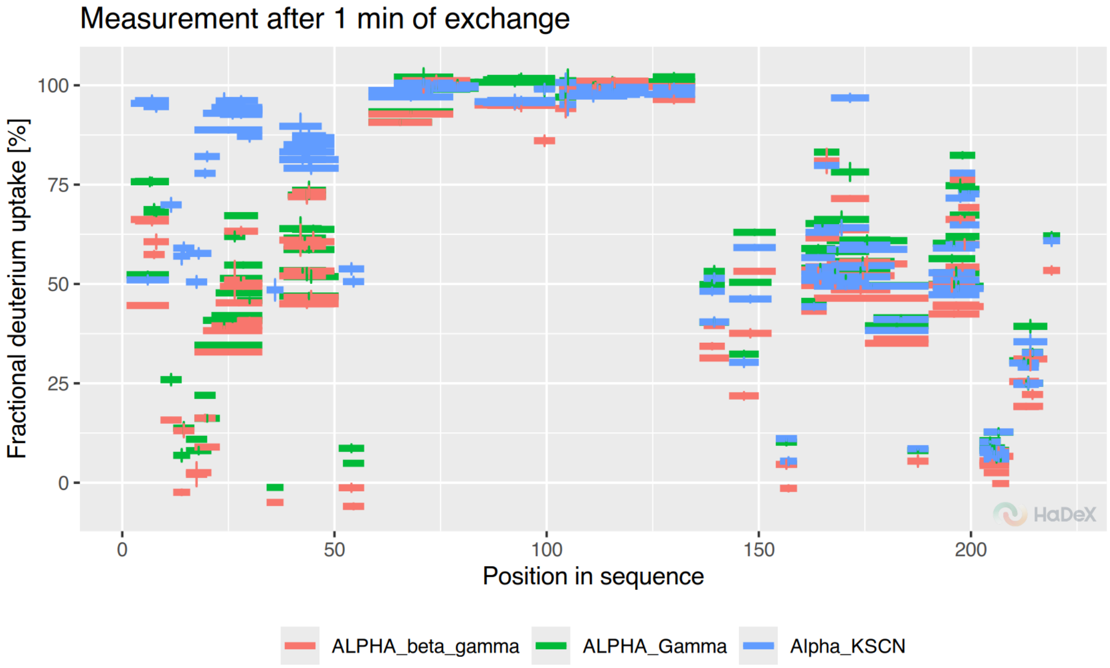

Pros:

- peptide length and position
- comparison of multiple biological states
- region with difference is easy to spot on
- uncertainty for each peptide

Plot variants:

- fractional/absolute values
- fractional values calculated with respect of theoretical/experimental maximal uptake
- plots with different time points can be plotted next to each other
- tooltips available in GUI

6.3 Woods plot

Woods plot presents the deuterium uptake difference between two biological states for the peptides. The results are presented with respect to the length of the peptide and its position in the protein sequence for a

given time point of the measurement. The statistical test (described in the subsection 4.2.1) is applied to determine the confidence limits values at the chosen level.

Example On the Woods plot below, we see fractional deuterium uptake difference between two biological states eEF1Bα and eEF1Bα in presence of eEF1Bγ for protein eEF1B The confidence limits indicate which differences are statistically significant at levels 98%.

```
calculate_diff_uptake(alpha_dat, states = c(states[3], states[1])) %>%
  plot_differential(., fractional = TRUE, show_houde_interval = TRUE) +
  labs(x = "Position in seqence",
       y = "Fractional deuterium uptake difference [%]",
       title = "Measurement after 1 min of uptake")
```

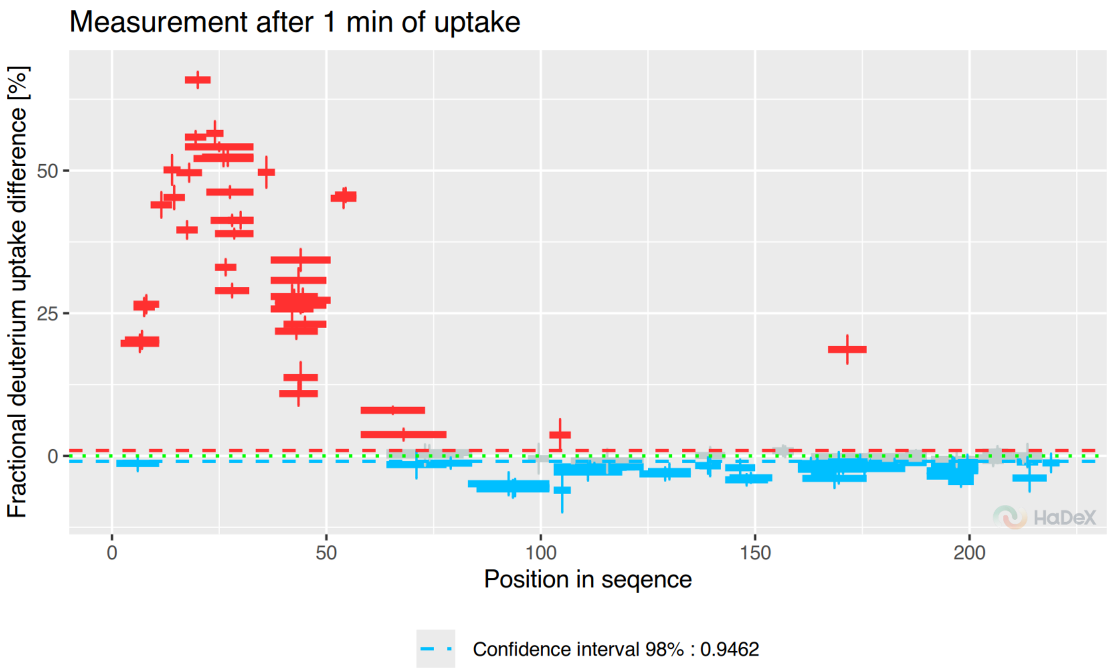

Pros:

- differential analysis between two states
- peptide length and position
- region with difference is easy to spot on
- uncertainty for each peptide
- hybrid statistical testing
- statistical level

Plot variables:

- fractional/absolute values
- fractional values calculated with respect of theoretical/experimental maximal uptake
- plots with different time points can be plotted next to each other
- peptides classified as statistically insignificant can be hidden
- tooltips available in GUI

6.4 Butterfly plot

Butterfly plot presents the deuterium uptake for all peptides in a given state at different time points at once. Each time point of measurement is indicated by a different color. Peptides are identified by their ID (peptides are numbered arranged by the start position).

Example Below, on the butterfly plot, we see how the deuterium uptake changes in time for state eEF1Bα for protein eEF1B. We see the different exchange speed - for some peptides, the change is stable in time, and for some peptides, there is no visible change in time.

```
create_state_uptake_dataset(alpha_dat, state = states[3]) %>%
  plot_butterfly(., fractional = FALSE)
```

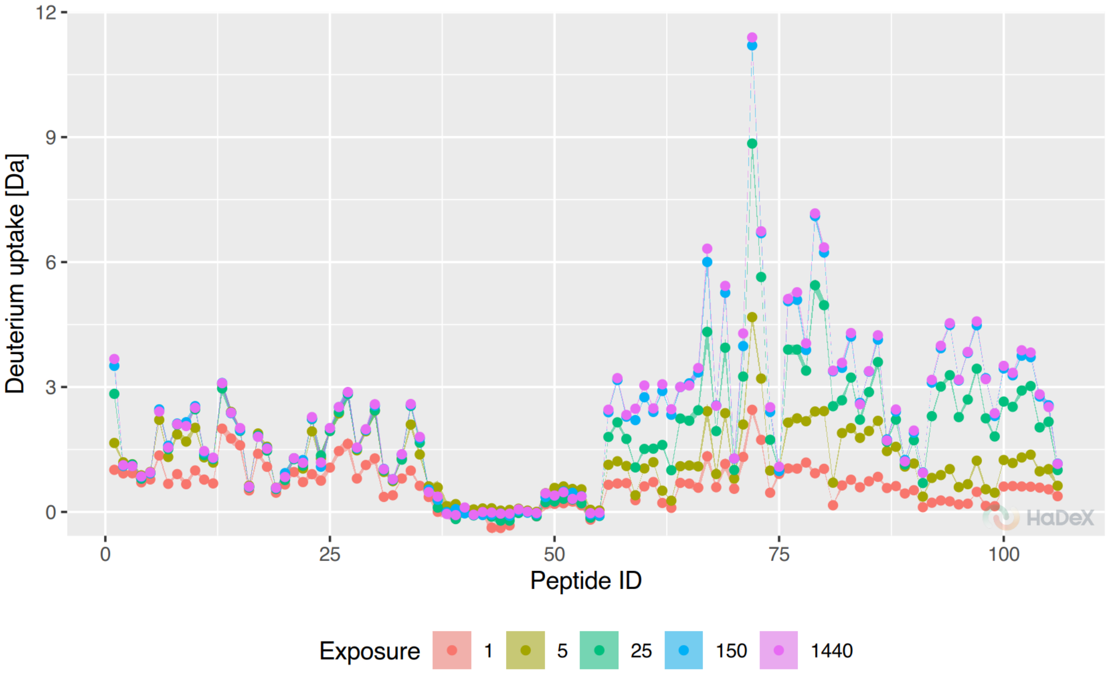

Pros:

- values for time course
- uncertainty for each peptide

Cons:

- only one state
- lost information of length and position of the peptide
- may be difficult to read

Plot variants:

- fractional/absolute values
- fractional values calculated with respect of theoretical/experimental maximal uptake
- different methods of showing uncertainty: bars or ribbons
- selected time points shown
- tooltips available in GUI

6.5 Butterfly differential plot

Butterfly differential plot shows the deuterium uptake difference between two biological states in the form of a butterfly plot. It shows the results for a peptide ID (peptides are numbered arranged by the start position). The results are shown for different time points at once (time points of measurement are indicated by the color).

Example Below, we see how the fractional deuterium uptake difference between states eEF1Bα and eEF1Bα in presence of eEF1Bγ changes over time. We see that for some peptides, the difference is smaller with time - perhaps because of the back exchange.

The measurements for 1440 min are hidden, as they are close to 0, as expected.

```
create_diff_uptake_dataset(alpha_dat, state_1 = states[3], state_2 = states[1]) %>%
  filter(Exposure < 1440) %>%
  plot_differential_butterfly(fractional = TRUE, show_houde_interval = TRUE)
```

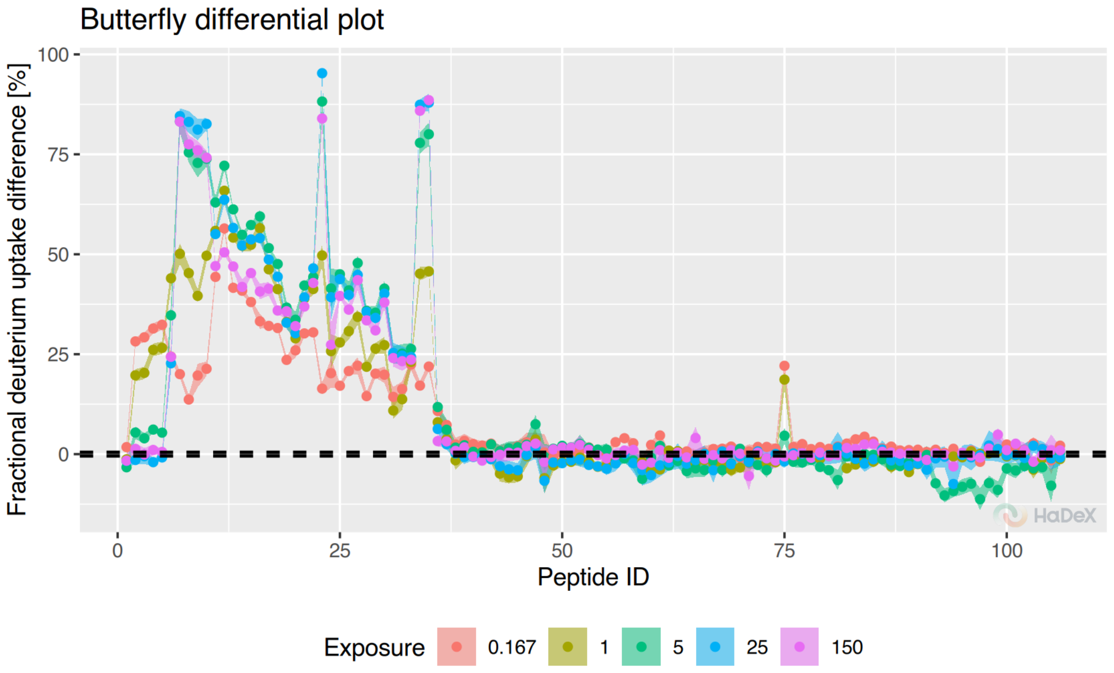

Pros:

- differential analysis between two states
- hybrid statistical testing
- statistical significance level
- values for time course
- uncertainty for each peptide

Cons:

- lost information of length and position of the peptide
- may be difficult to read

Plot variants:

- fractional/absolute values
- fractional values calculated with respect of theoretical/experimental maximal uptake
- different methods of showing uncertainty: bars or ribbons
- selected time points shown
- tooltips available in GUI

6.6 Chiclet plot

Chiclet plot shows the fractional deuterium uptake in the form of a heatmap for the peptides in a given biological state. One tile indicates the peptide (identified by its ID - number arranged by the start position) in a time point of measurement. The color of the tile indicates the fractional deuterium uptake (according to the legend below the plot).

Example In the chiclet plot below, we can see the deuterium uptake values for peptides (indicated by their ID) in state eEF1Bα during the time course of the experiment. The cross symbols indicate the uncertainty of the measurement (the bigger the cross sign, the bigger the uncertainty).

```
create_state_uptake_dataset(alpha_dat, state = states[3]) %>%
  filter(Exposure < 1440) %>%
  plot_chiclet(show_uncertainty = TRUE, fractional = FALSE)
```

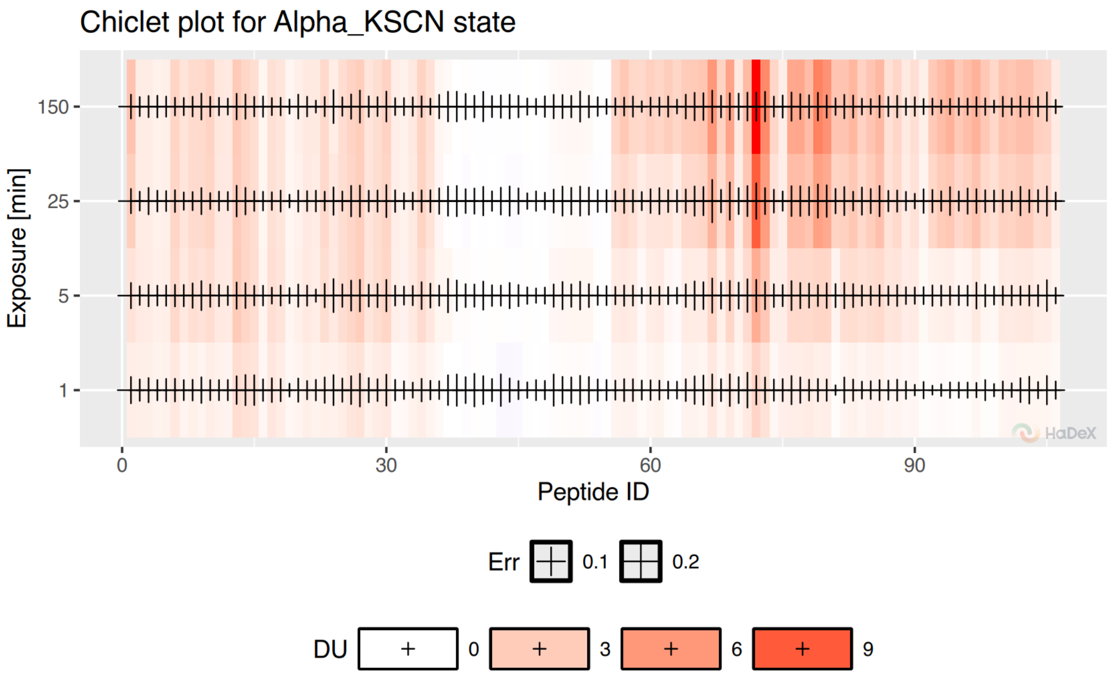

Pros:

- values for time course
- uncertainty for each peptide

Cons:

- only one state

- lost information of length and position of the peptide
- may be difficult to read
- small changes in values may be difficult to spot

Plot variants:

- fractional/absolute values
- fractional values calculated with respect of theoretical/experimental maximal uptake
- uncertainty can be hidden to improve the readability of the plot
- peptides classified as statistically significant can be hidden
- selected time points shown
- tooltips available in GUI

6.7 Chiclet differential plot

Chiclet differential plot shows the deuterium uptake difference between two biological states in the form of a heatmap. One tile indicates the peptide (identified by its ID - number arranged by the start position) in a time point of measurement. The color of the tile indicates the deuterium uptake difference (according to the legend below the plot).

Example On the chiclet differential plot below, we see the fractional deuterium uptake difference between states eEF1Bα and eEF1Bα in presence of eEF1Bγ for protein eEF1B. We see that some peptides are protected (red), and some are deprotected (blue). The cross symbols indicate the uncertainty of the measurement (the bigger the cross sign, the bigger the uncertainty).

```
diff_uptake_dat %>%
  filter(Exposure < 1440 & Exposure > 0.001) %>%
  plot_differential_chiclet(show_uncertainty = TRUE, fractional = TRUE)
```

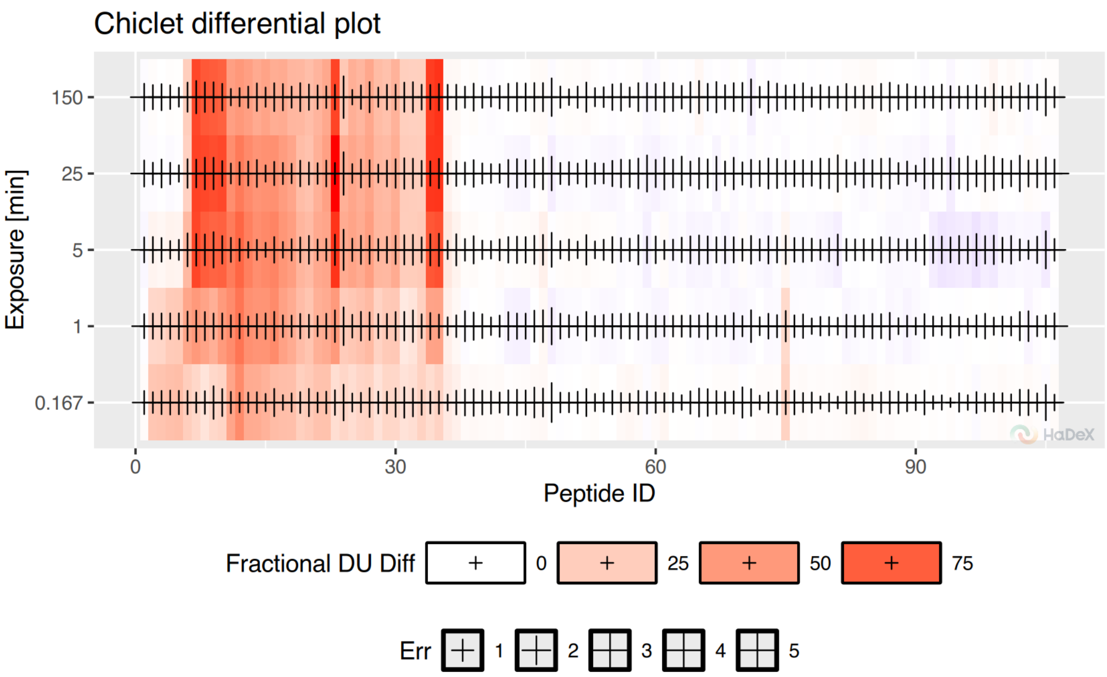

Pros:

- differential analysis between two states
- values for time course
- uncertainty for each peptide

Cons:

- lost information of length and position of the peptide
- may be difficult to read
- small changes in values may be difficult to spot

Plot variants:

- fractional/absolute values
- fractional values calculated with respect of theoretical/experimental maximal uptake
- uncertainty can be hidden to improve the readability of the plot
- peptides classified as statistically significant can be hidden
- selected time points shown
- tooltips available in GUI

6.8 Volcano plot

The volcano plot shows the deuterium uptake difference for two biological states for peptide and its p-value for double testing on statistical significance (Hageman and Weis 2019). On the x-axis, there is a deuterium uptake difference with its uncertainty (combined and propagated). On the y-axis, there is a P-value calculated for each peptide in a specific time point of a measurement as a un-paired t-test on given significance level (on mass measurement from the replicates to indicate if the measured mean is significantly different between two states, as the deuterium uptake difference between states can be rewritten as

$$\Delta D = D_A - D_B = m_{t,A} - m_0 - (m_{t,B} - m_0) = m_{t,A} - m_{t,B}$$

for states A and B. The values of deuterium uptake difference from all time points are shown on the plot.

The dotted red lines indicate confidence limits for the values. The horizontal line indicates the confidence limit based on chosen confidence level to give a threshold on a P-value. The vertical lines indicate the confidence limit from Houde test for all time points and indicate a threshold on deuterium uptake difference. The statistically significant points are in the top left and right corners of the plot.

Example On the volcano plot below, we see the results for deuterium uptake difference between states eEF1Bα and eEF1Bα in presence of eEF1Bγ for protein eEF1B in all time points. The points in the left and right upper corner are statistically significant using the hybrid testing.

```
p_dat <- create_p_diff_uptake_dataset(alpha_dat)

plot_volcano(p_dat, show_confidence_limits = TRUE)
```

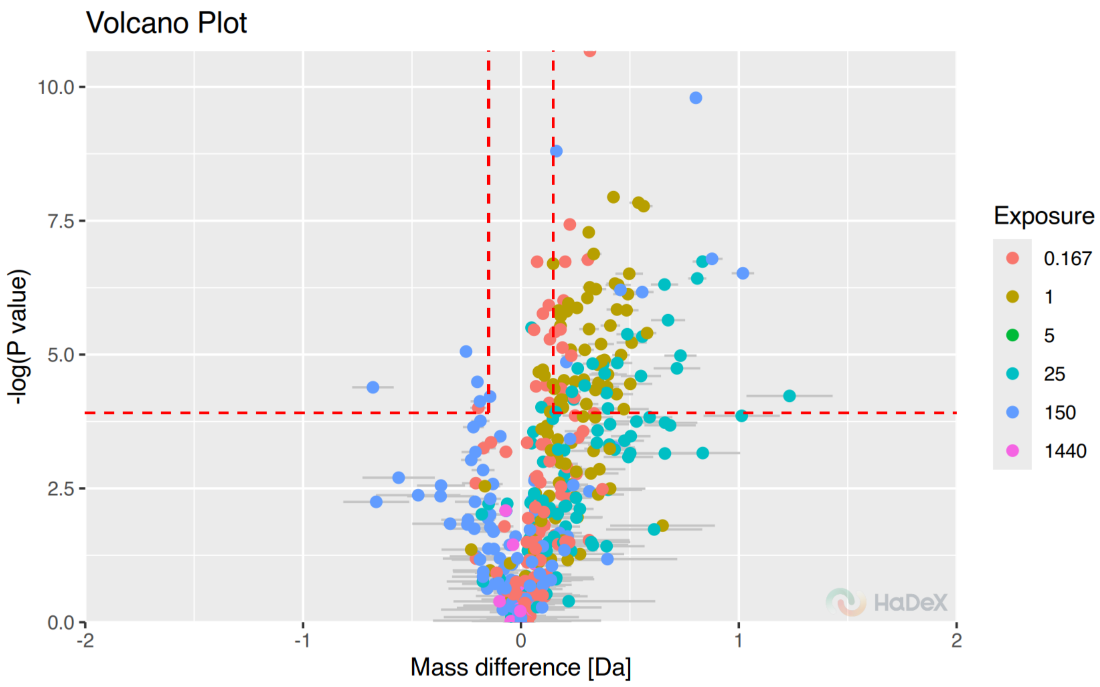

Pros:

- hybrid statistic test
- time course information
- uncertainty

Cons:

- peptide length and position lost
- no information of region

Plot variations:

- selected time points
- absolute/fractional values s
- hidden insignificant values
- tooltips available in GUI

6.9 Uptake curve

Uptake curves show the changes in exchange in time for a specific peptide for its state.

Example On the uptake curve below, we see how the exchange goes for peptide GFGDLKSPAGL in all three states for protein eEF1Ba.

```
calculate_peptide_kinetics(dat = alpha_dat) %>%
plot_uptake_curve() +
  ylim(c(0, NA))
```

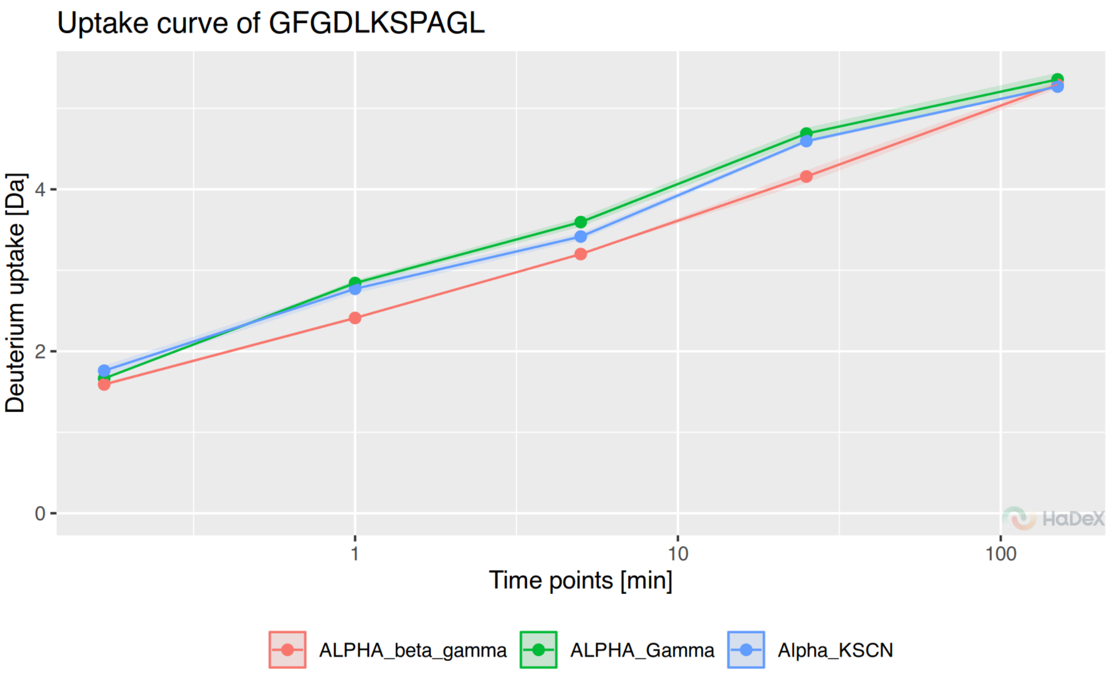

Pros:

- time course
- state comparison
- change of uptake tendency visible
- focus on one peptide
- uncertainty

Plot variations:

- fractional/absolute values
- fractional values calculated with respect of theoretical/experimental maximal uptake
- selected states shown
- different methods of showing uncertainty: bars or ribbons
- tooltips available in GUI

6.10 Uncertainty plot

Uncertainty plot is new visualization method, showing the uncertainty of measurement of deuterium uptake for peptides to spot the regions where the uncertainty is higher. This plot may be used as a quality control of the experiment, as discussed in the subsection @ref(#mvp). The presented uncertainty is in Daltons, making the threshold 1 Da as proposed limit of acceptance.

Example The plot below presents uncertainty values for multiple time points for state eEF1Bα. We see that the uncertainty is relatively low for all the measurement, and none of the value is suspicious.

```
alpha_dat %>%
  filter(Exposure > 0) %>%
  plot_uncertainty()
```

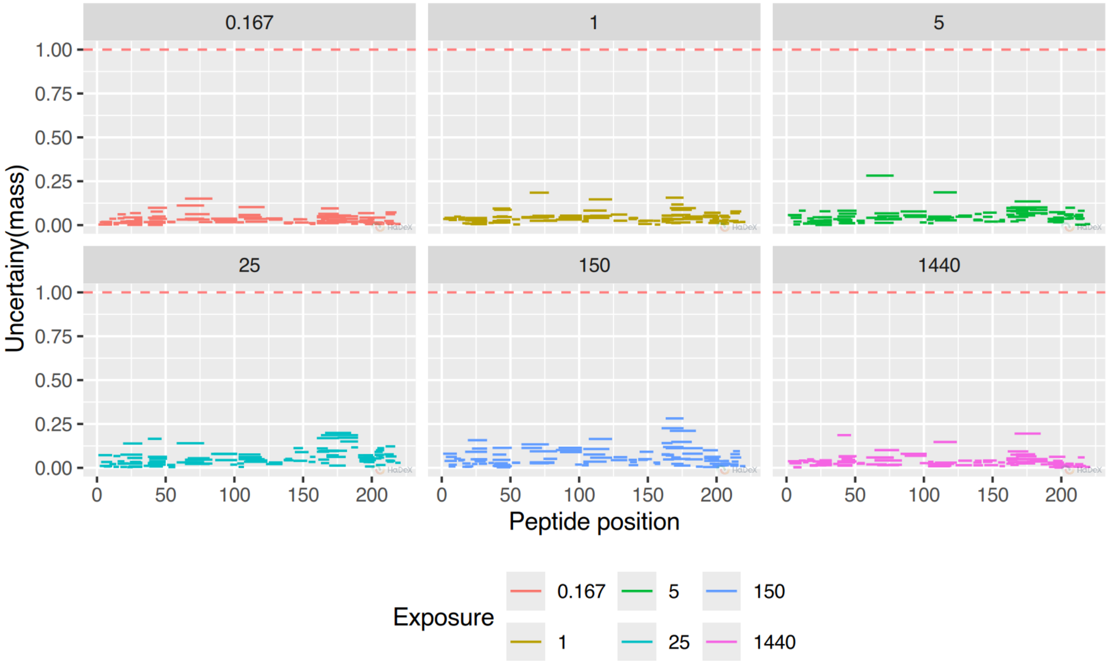

Pros:

- experimental quality control
- regions with high measurement uncertainty are easy to spot
- peptide length and position
- region with difference is easy to spot on

Plot variants:

- plot for only one time point can be plotted
- selected time points shown
- threshold can be hidden
- first amino omitted
- tooltips available in GUI

6.11 Manhattan plot

Manhattan plot is a novel plot, presenting the P-value of statistical significance between two states.

Example In this example, we present the P values of difference between two biological states: eEF1Bα and eEF1Bα in presence of eEF1Bγ. We can see the regions where the difference is statistically significant - above the significance level (detailed as option in create_p_diff_uptake_dataset, or by default 0.98).

```
p_diff_dat <- create_p_diff_uptake_dataset(dat = alpha_dat, diff_uptake_dat = diff_uptake_dat,
                                           state_1 = states[3], state_2 = states[1])
plot_manhattan(p_diff_dat, show_peptide_position  = TRUE)
```

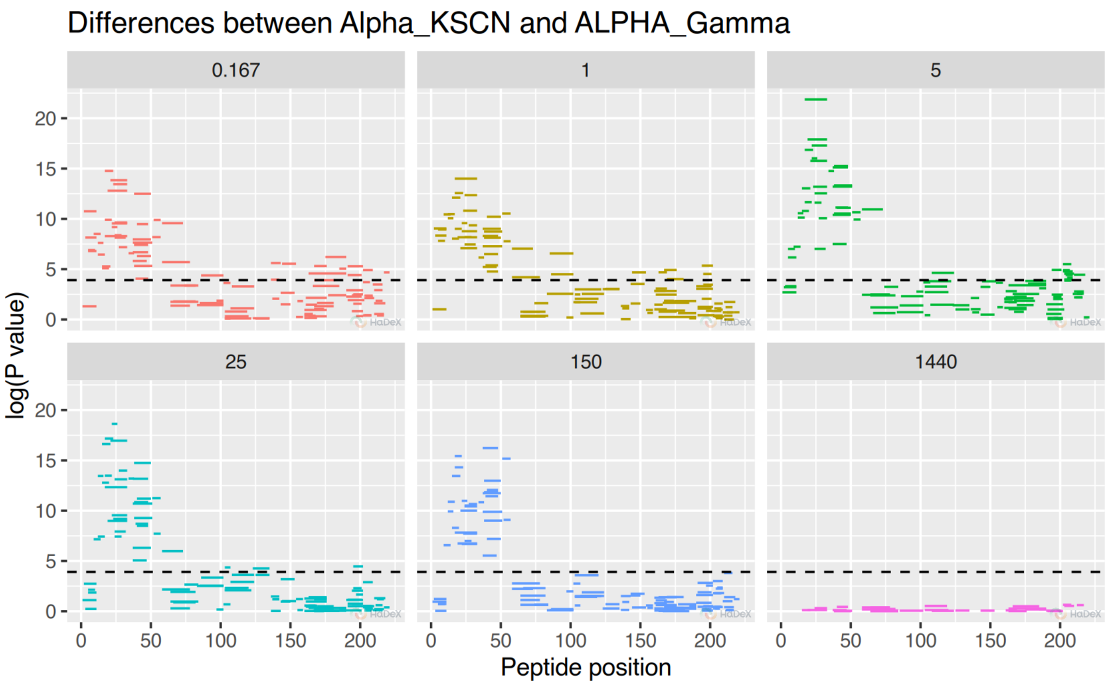

Pros:

- peptide length and position
- shows significant regions
- statistical interval shown

Plot variations:

- plot for only one time point can be plotted
- selected time points shown
- threshold can be hidden
- first amino omitted
- tooltips available in GUI

6.12 High-resolution plot

The biggest limitation of previous methods of deuterium uptake visualization is that the results are on the peptide level. However, we offer a method of deuterium uptake averaging from peptide level into high-resolution level, using the weighted method of averaging (as described in the section 3.4). Then, the results are presented on the heatmap.

Example

```
kin_dat <- create_uptake_dataset(alpha_dat, states = "Alpha_KSCN")
aggregated_dat <- create_aggregated_uptake_dataset(kin_dat)
plot_aggregated_uptake(aggregated_dat)
```

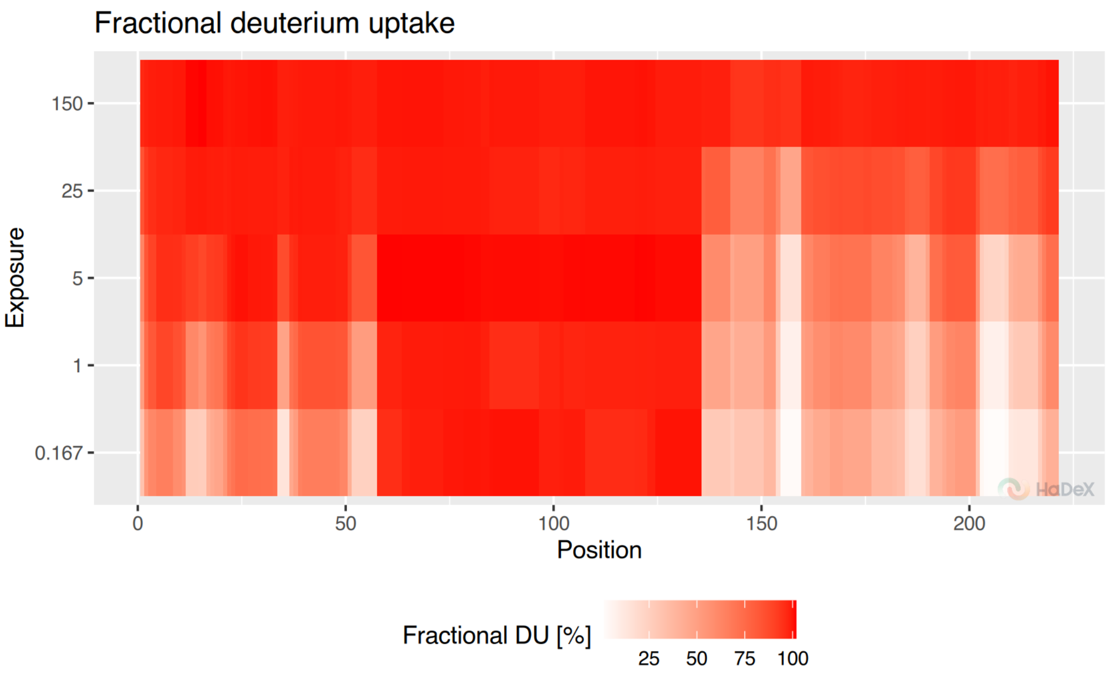

Pros:

- high-resolution
- time course
- uncertainty of averaged values

Cons:

- values are an approximation
- linear presentation lacking conformational information

Plot variations:

- fractional/absolute values
- fractional values calculated with respect of theoretical/experimental maximal uptake
- plot divided into panels
- tooltips available in GUI

6.13 Differential High-resolution plot

As most of our methods of visualization, also the high-resolution plot has its differential version. The plot presents averaged uptake difference values using weighted approach.

Example On the structure, we see the fractional deuterium uptake difference after 25 minutes between states eEF1Bα and eEF1Bα in presence of eEF1Bγ for protein eEF1B. We see that some peptides are protected (red), and some are deprotected (blue).

```
diff_uptake_dat <- create_diff_uptake_dataset(alpha_dat, state_1 = states[3], state_2 = states[1])
averaged_diff_dat <- create_aggregated_diff_uptake_dataset(diff_uptake_dat)
plot_aggregated_differential_uptake(averaged_diff_dat, panels = FALSE)
```

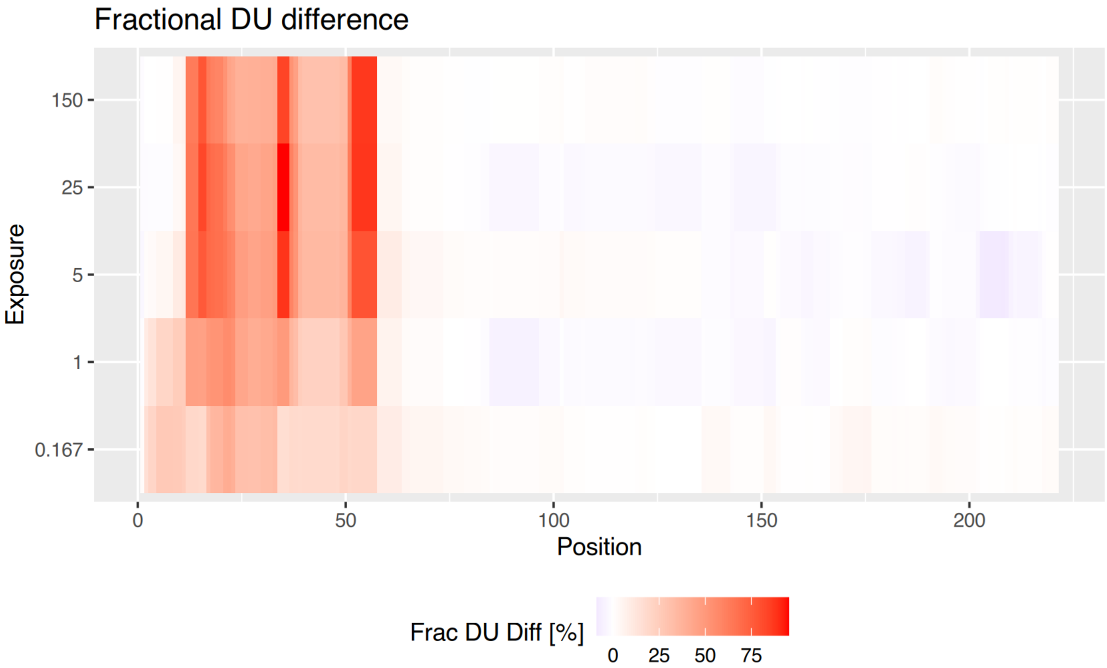

Pros:

- high-resolution
- comparative analysis
- time course
- uncertainty of averaged values

Cons:

- values are an approximation

Plot variations:

- fractional/absolute values
- fractional values calculated with respect of theoretical/experimental maximal uptake
- plot divided into panels
- tooltips available in GUI

6.14 High-resolution on 3D structure

High-resolution values not only can be presented linearly on high-resolution plot (as above), but also on 3D structure, if available. This way, the calculated values are connected with spatial aspect. This option is available both for single state uptake and differential uptake.

Example Structure below is mapped with deuterium uptake values after 1 minute for eEF1Bα state. Values are averaged using weighted approach, color signifies no exchange (white) to high exchange (red).

```
pdb_file_path <- system.file(package = "HaDeX2", "HaDeX/data/Model_eEF1Balpha.pdb")

plot_aggregated_uptake_structure(aggregated_dat,
                                 differential = FALSE,
                                 time_t = 1,
                                 pdb_file_path = pdb_file_path)
```

Pros:

- averaged values
- spatial information
- spinning, rotating and zooming options

Cons:

- only one time point at a time
- no personalization
- no information on numerical uptake values
- no legend

Plot variations:

- fractional/absolute values
- fractional values calculated with respect of theoretical/experimental maximal uptake

6.15 Coverage heatmap

Coverage heatmap plot is a variation of standard coverage plot - but with each peptide is colored to signal specific value. This plot is particularly useful when presenting AUC (Area Under the Curve, defined in the subsection 3.6) or back-exchange values, as they are specified for peptide uptake curve.

Example Plot below presents the AUC values for eEF1Bα. We see that for the majority of regions the AUC values is close to 1, signifying fast exchange with exception for one strong region and small sub-regions.

```
auc_dat <- calculate_auc(create_uptake_dataset(alpha_dat))
plot_coverage_heatmap(auc_dat, value = "auc")
```

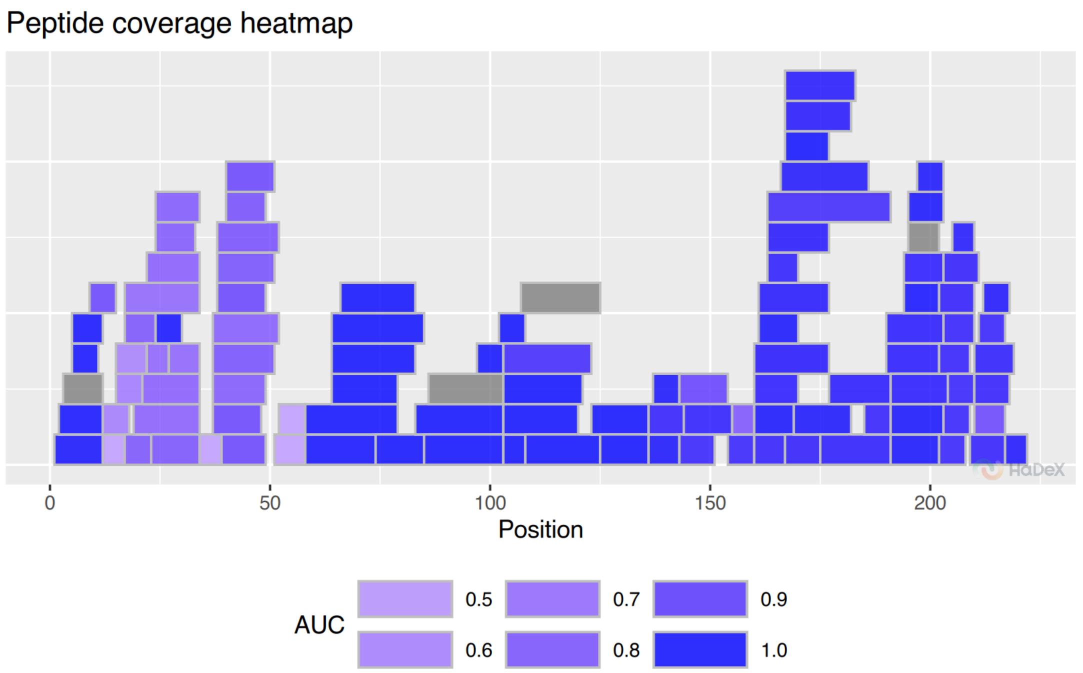

Each of the AUC value for different biological state is calculated separately. In order to meaningfully compare AUC values, we must select the same fully deuterated control for all of them - to have the same reference point of 100 % exchange. Otherwise, when the uptake curves have different values in the plus infinity, the AUC values described only the speed of the exchange taken separately. In the end, this value is more useful when used to compare uptake curves for a specific peptide under different biological conditions.

Example The coverage heatmap plot below presents the back-exchange values for peptides form eEF1Bα. Back-exchange is believed to be on average close to 30%, as we see on the plot. Some peptides - especially shorter ones - have grater back-exchange.

```
bex_dat <- calculate_back_exchange(alpha_dat, state = "Alpha_KSCN")
plot_coverage_heatmap(bex_dat, value = "back_exchange")
```

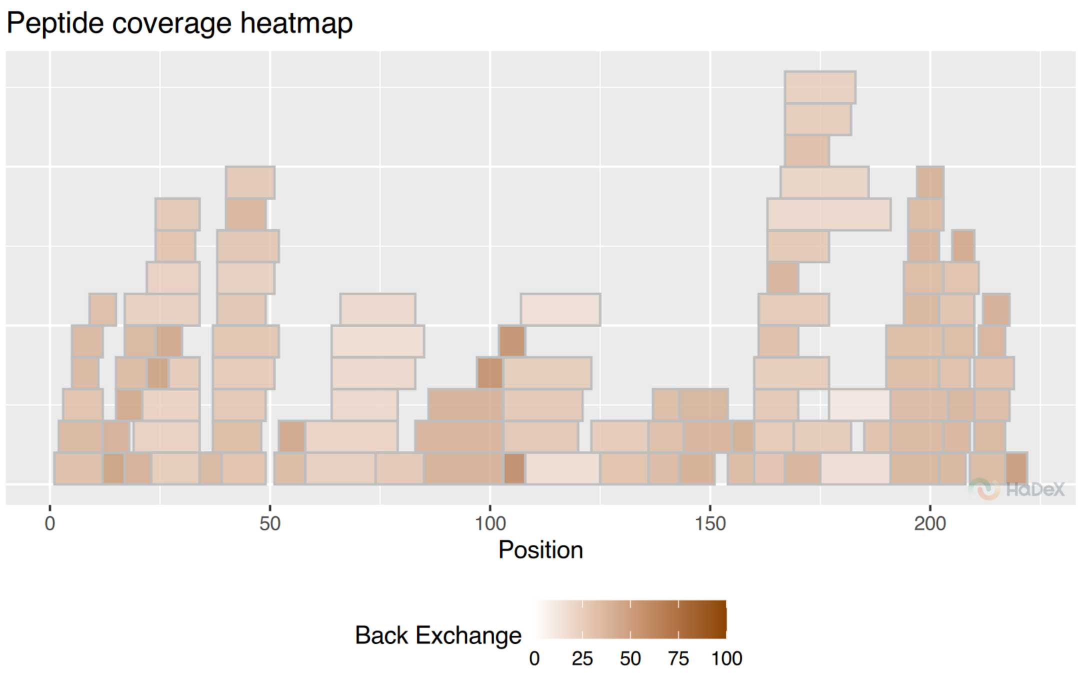

Pros:

- auc / back-exchange values
- one value for whole time course
- peptide length and position

Cons:

- values may be hard to interpret within small range

Plot variations:

- tooltips available in GUI

6.16 Summary of the uptake plots

Below we compare the aspects of the plots.

types	time course	length of the peptide	uncertainty	all peptides	different states	position
comparison	FALSE	TRUE	TRUE	TRUE	TRUE	TRUE
Woods (differential)	FALSE	TRUE	TRUE	TRUE	TRUE	TRUE
butterfly	TRUE	FALSE	TRUE	TRUE	FALSE	FALSE
butterfly differential	TRUE	FALSE	TRUE	TRUE	TRUE	FALSE
volcano	TRUE	FALSE	TRUE	TRUE	TRUE	FALSE
chiclet	TRUE	FALSE	TRUE	TRUE	FALSE	FALSE
chiclet differential	TRUE	FALSE	TRUE	TRUE	TRUE	FALSE
uptake curve	TRUE	FALSE	TRUE	FALSE	TRUE	FALSE

The columns indicate:

- time course - does this plot show the results from different time points?
- length of the peptide - does the plot show the information of the length of the peptide?
- uncertainty - does this plot show the uncertainty of the measurement?
- all peptides - does this plot show the results for all of the peptides?
- different states - does this plot show the results for different states?

7 Example HDX-MS data analysis

7.1 Introduction

This document summarizes functions of HaDeX2 web server by describing example data analysis. The example dataset we will use here is part of a previously published dataset on the SecA ATPase protein (Krishnamurthy et al. 2021). SecA is an essential part of the bacterial Sec protein secretion system where it interacts with secretory client proteins as well as its associated cytoplasmic chaperones and is ultimately targeted to the SecYEG membrane channel where it interacts with both non-folded clients as well as the export channel, thereby regulating or powering protein export.

SecA is a DEAD motor domain ATPase, and in the cytoplasm the protein predominantly exists as a dimeric, ADP bound 'quiescent' state. Only through a series of interactions with the translocate and secretory clients does SecA become fully activated and reaches the actively ATP hydrolysing monomeric state, functioning as a molecular motor, driving protein translocation.

7.2 Data analysis with HaDeX2

In the next sections we will take the SecA example data and stepwise run it though the analysis pipeline of the HaDeX2 web server. In this example we focus on SecA in its dimeric state, and we compare how binding to ADP, switching the protein to its quiescent state, affects the structural dynamics of SecA.

7.2.1 Input Data

The first step in our HDX-MS data analysis is to upload the data. In the HaDeX2 webserver, in the 'Input data' tab, we can upload the data as DynamX 3.0 'cluster' files. Click 'Browse' to select the example data file `inst/HaDeX/data/SecA_cluster_wt_ADP.csv`, after which the 'File status' should report the file is valid and is of the DynamX3.0 format. For this dataset, we also have a structure file available, `inst/HaDeX/data/SecA_monomer.pdb` (modified from RCSB PDB entry 2VDA), and it can be uploaded below in a similar fashion.

Next, we set the parameters for the analysis of this data. We select the correct protein ('Accession'), then choose which peptides correspond to the Maximal exchange control (Accession | Full Deuteration control | 0.167). The `No deuterated` time point is set to 0.

7.2.2 Deuterium uptake

We can start by looking at deuterium uptake per peptide/time point. The first plot generated is the Deuterium uptake plot (Figure 2). This shows replicate-averaged deuterium uptake values per peptide, for all selected states, at a given time point. It provides a good starting point to inspect the data, and verify no peptide deuterium uptake exceeds the maximal uptake control uptake.

The second plot on this page is the Woods plot. This plot shows the deuterium uptake difference between two selected states, in our case this is a comparison between SecA dimer WT and SecA dimer + ADP. From the generated figure (Figure 3, we can immediately see that two regions stand out, both part of the SecA nucleotide-binding cleft, the first around 102-114 (Helicase motif I, NBD1) and the second at 410-428 (NBD

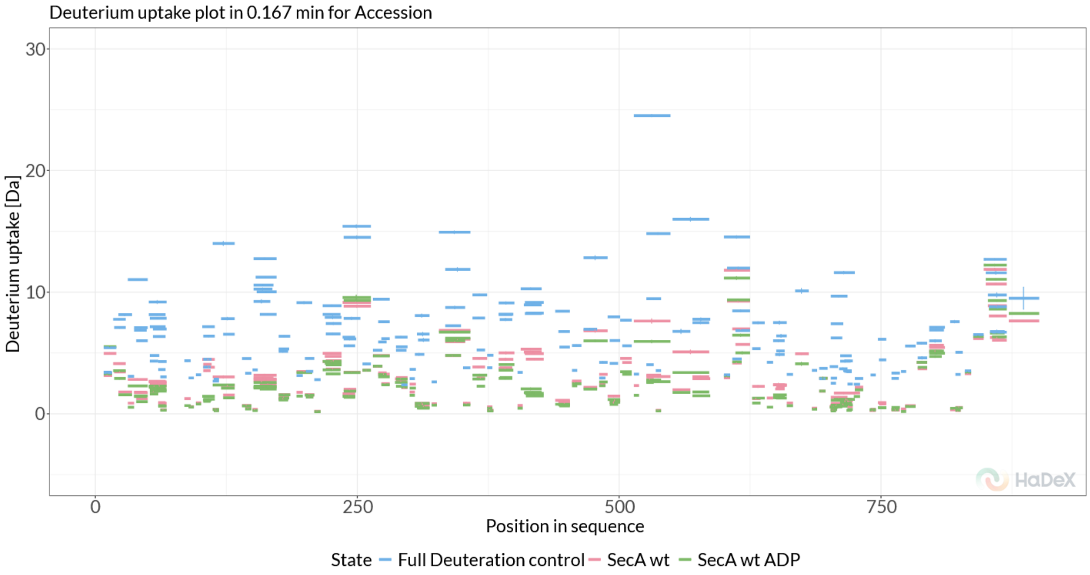

Figure 2: Deuterium uptake plot.

linker region). Deuterium uptake differences in these regions are positive (red), indicating that these regions rigidify upon ADP binding, and this rigidification allosterically propagates to SecA's client-binding clamp, thereby regulating its client interactions and activity.

If we want to inspect these regions in more detail, we can use the 'Ranges' section in the sidebar to zoom the x range to the regions of interest.

Next, to explore the data further, we can switch the plot to 'Fractional uptake', where we use the maximal exchange control as reference. Select the checkbox 'Calculation > Fractional values' and then at 'time points' make sure to select 'chosen control' for 'Deut 100% Exposure'. With these settings the Woods plot shows the differential HDX data in terms of fractional deuterium uptake differences, removing the bias of peptide length. Again, we see the same two regions stand out, and if we explore more time points ('time points > Measurement Exposure > 2'), we find additional peptides which show rigidification, such as 570-587 (motif VI, NBD2) and 630-645 (scaffold domain).

The 'Butterfly Differential plot' provides a more comprehensive view of all time points simultaneously. Switching to this view, we can more easily track the evolution of deuterium uptake differences over time and therefore all four regions of interest are clearly visible (Figure 4). This view reviews another potentially rigidified region, showing only at later timepoints, around the region 772-782.

The 'Chiclet Plot' provides a direct overview of the time-evolution of differentially exchanged regions. Switching the plot to 'Fractional Values', we can see that two regions (102-114, 410-428) are present independent of the time point, while the differences in 570-587 increase over time, and the 630-645 regions shows a 'appear-disappear' behavior, being most prominent at intermediate time points (Figure 5).

To zoom in on this phenomenon, we can view the uptake curve of a single peptide using the 'Uptake Curves Plot / Differential Plot'. A single peptide can be selected in the "Peptide" section of the sidebar. Filter for sequence 'AIANAQRKVE', and choose this particular peptide from the filtered table. Now, we can confirm that indeed for this peptide, SecA WT initially takes up deuterium faster compared to SecA bound to ADP, but at later time points the curves converge. The 'Differential Plot' directly shows the differences as a rise and decay.

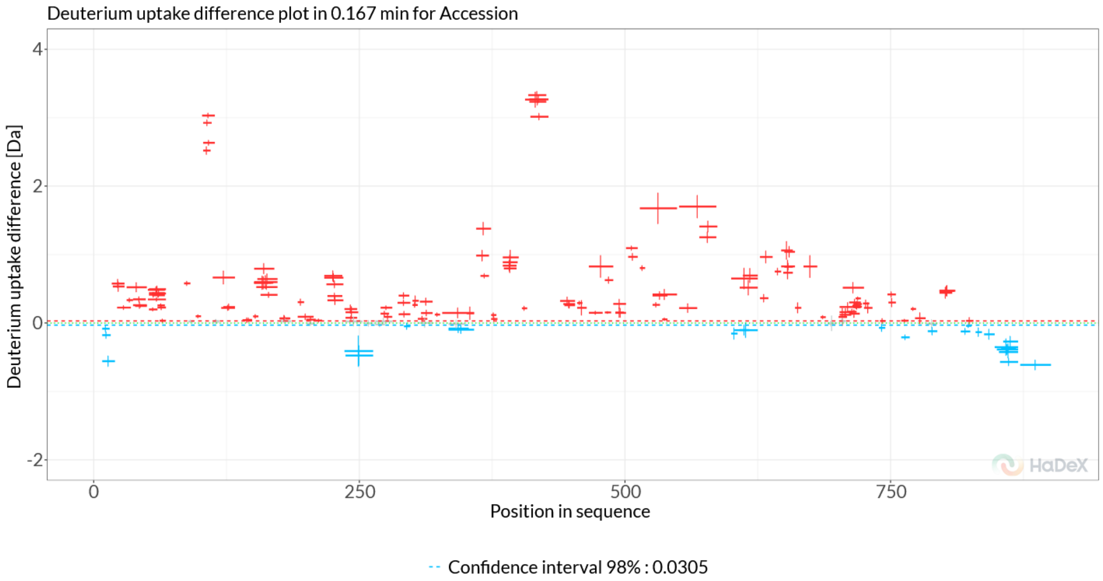

Figure 3: Woods plot

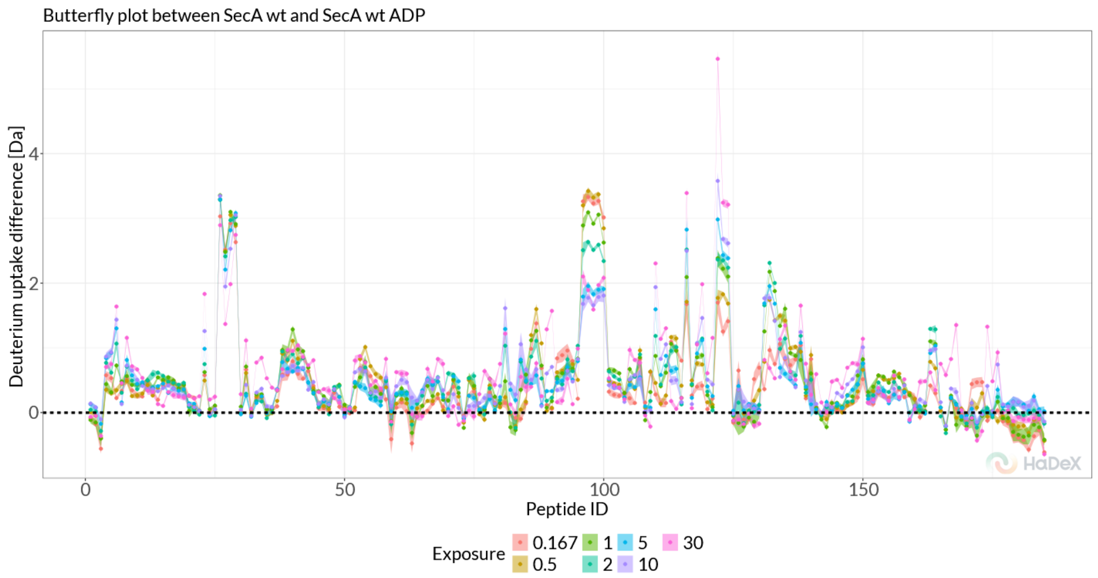

Figure 4: Butterfly Differential plot.

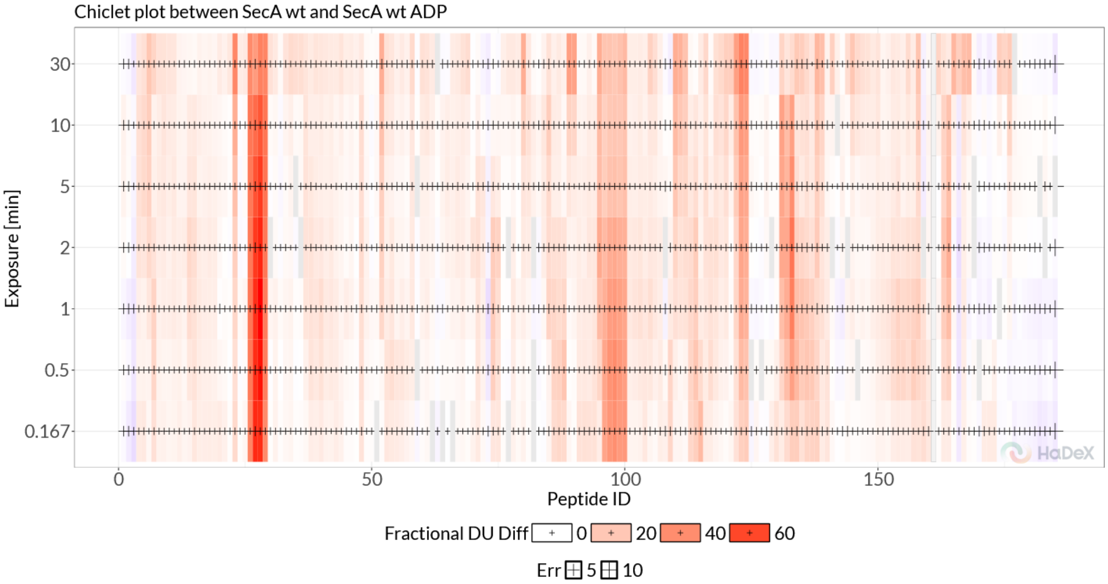

Figure 5: Chiclet Plot.

7.2.3 Time-based data

The 'Time-based data' tab provides more information on replicates and statistical significance between states. The 'Replicates' view gives a quick overview of the replicates for each state and time point. For example, the second plot on this page shows directly that for the non-deuterated control (0s), there are a couple of peptides where one or more replicates are missing. We can use this information to quickly identify peptides which might need to be excluded or we need to collect additional data. The Manhattan plot provides similar information the Woods plot, but instead of reporting on deuterium uptake differences, it shows the statistical significance of these differences. This can be an important tool when judging how to interpret deuterium uptake differences between peptides. In our example data, the Manhattan plot shows that generally we have obtained a lot of peptide with statistically significant differences, where regions with low or no differences in D-uptake also show low statistical significance. Finally, in the uncertainty plot we can see the calculated standard error of the mean for the replicates to indicate regions of higher measurement uncertainty for optional double-check, part of quality control.

7.2.4 Hi-res + 3D

Because we have provided a structural model alongside our peptide data, HaDeX2 allows us to directly visualize residue-level data mapped onto the structure. To achieve this, we can use 'Differential Heatmap + 3D Vis'. Again, we use 'Fractional values', and set 'Deut 100% Exposure' to 'chosen control'. The generated heatmap (Figure 6) shows the same regions of interest, with as critical difference that now instead of looking at peptide-level, the data has been mapped to individual residues by weighted averaging.

In the differential heatmap, we can now see our regions of interest with residue-level resolution. The two main regions, 102-114 and 410-428, are clearly visible, as well as the more subtle differences in 570-587 and 630-645. The region around 772-782 is less clear in this view, likely because this region is only subtly affected and only at later time points.

The 3D viewer provides an interactive way to explore these differences in the context of the protein structure. By mapping the differential HDX data onto the 3D structure of SecA, we can directly see how ADP binding rigidifies specific regions of the protein (Figure 7). In this figure, we can clearly see the strongest response in helicase motif I, rigidifying upon ADP binding, as well as the allosteric propagation of this effect through the scaffold regions ultimately resulting in a long-range allosteric response in the client-binding PPD domain.

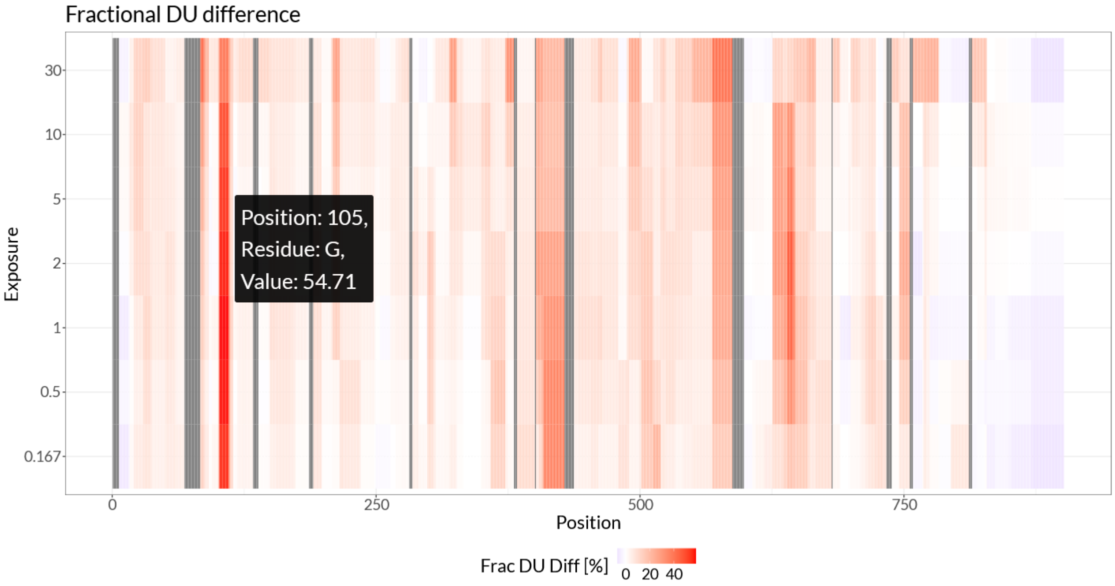

Figure 6: Differential hcheatmap.

For more control over the visualization colors and plot ranges, the residue-level data can be exported in a .csv format and subsequently visualized by HDX-Viewer (Bouyssié et al. 2019). When exporting the data to HDX-Viewer, click the 'Export data for HDXViewer' and follow the steps described in the popup window.

7.2.5 Measurements

The 'Measurements' tab allows us to further inspect the individual replicate measurement results. This tab provides a per-peptide view where we can use the peptide table in the sidebar to select peptides of interest. Then, for the selected exposure time, we can now inspect each replicate measurement, see how they are distributed and we see which charged states were used and how they compare. The second graphs shows this information for all time points as a peptide uptake curve.

7.2.6 Sequence data

The 'Sequence data' tab can be used to reconstruct the sequence from the uploaded data and shows the coverage and sequence length. This can be used as a quick sanity check to confirm the correct sequence information was used. This tab also provides peptide coverage plots. The 'Coverage' graph (Figure 8) gives a quick overview of which peptides were identified, and which regions have no coverage. The 'Position Frequency' graph on this page shows for each residue how many peptides cover this region, providing information on peptide redundancy.

7.2.7 Summary

The summary is a very convenient and quick way to generate a summary report of the uploaded data, according to community recommendations (Masson et al. 2019). The report can be exported in a variety of tabular formats.

Bondarchuk, Tetiana V, Vyacheslav F Shalak, Dmytro M Lozhko, Agnieszka Fatalska, Roman H Szczepanowski, Vladyslava Liudkovska, Oleksandr Yu Tsuvariev, Michal Dadlez, Anna V El'skaya, and Boris S Negrutskii.

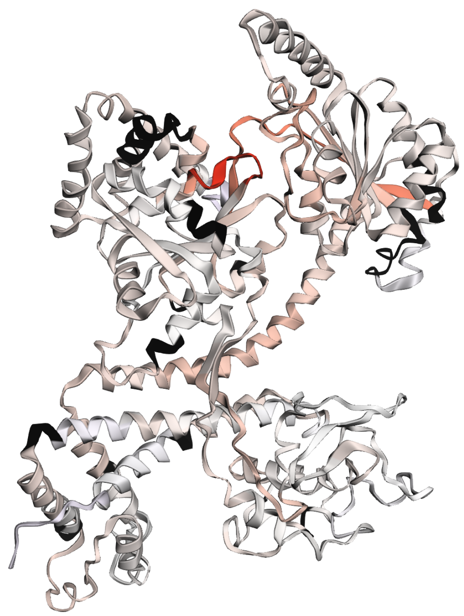

Figure 7: 3D view of differential HDX data on the structure of SecA.

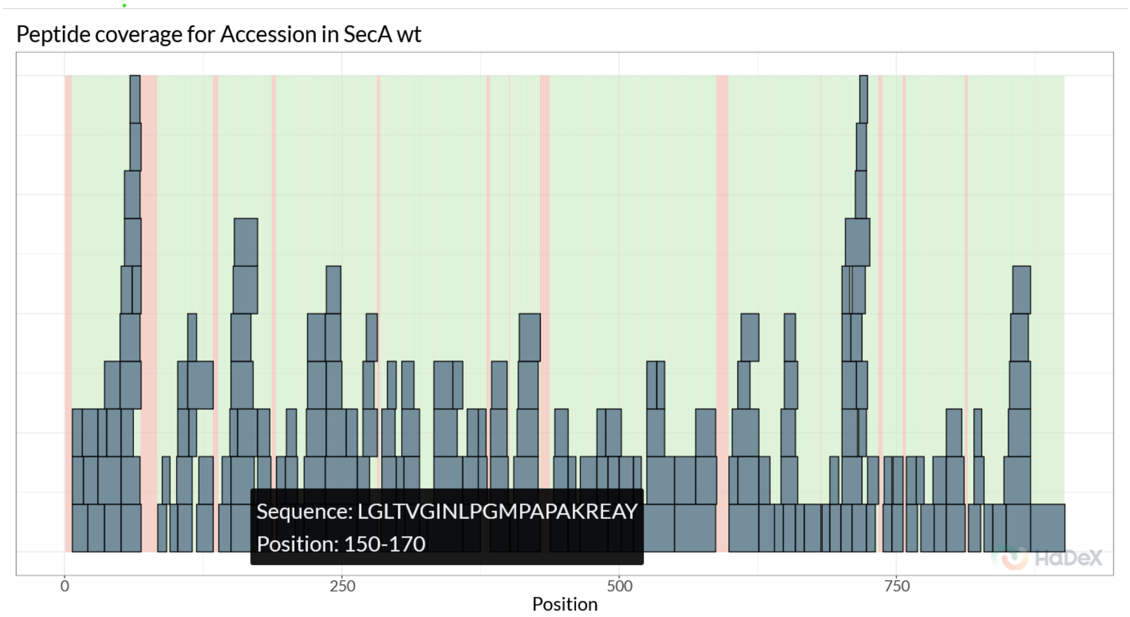

Figure 8: Peptide coverage for SecA WT.

2022. “Quaternary Organization of the Human eEF1B Complex Reveals Unique Multi-GEF Domain Assembly.” *Nucleic Acids Research* 50 (16): 9490–9504. https://doi.org/10.1093/nar/gkac685.

Bouyssié, David, Jean Lesne, Marie Locard-Paulet, Renaud Albigot, Odile Burlet-Schiltz, and Julien Marcoux. 2019. “HDX-Viewer: Interactive 3D Visualization of Hydrogen–Deuterium Exchange Data.” *Bioinformatics* 35 (24): 5331–33. https://doi.org/10.1093/bioinformatics/btz550.

Hageman, Tyler S., and David D. Weis. 2019. “Reliable Identification of Significant Differences in Differential Hydrogen Exchange-Mass Spectrometry Measurements Using a Hybrid Significance Testing Approach.” *Analytical Chemistry* 91 (13): 8008–16. https://doi.org/10.1021/acs.analchem.9b01325.

Houde, Damian, Steven A. Berkowitz, and John R. Engen. 2011. “The Utility of Hydrogen/Deuterium Exchange Mass Spectrometry in Biopharmaceutical Comparability Studies.” *Journal of Pharmaceutical Sciences* 100 (6): 2071–86. https://doi.org/10.1002/jps.22432.

Joint Committee for Guides in Metrology. 2008. “JCGM 100:2008 Evaluation of Measurement Data — Guide to the Expression of Uncertainty in Measurement.” Technical Report JCGM 100:2008. Joint Committee for Guides in Metrology.

Keppel, Theodore R., and David D. Weis. 2015. “Mapping Residual Structure in Intrinsically Disordered Proteins at Residue Resolution Using Millisecond Hydrogen/Deuterium Exchange and Residue Averaging.” *Journal of the American Society for Mass Spectrometry* 26 (4): 547–54. https://doi.org/10.1007/s13361-014-1033-6.

Krishnamurthy, Srinath, Nikolaos Eleftheriadis, Konstantina Karathanou, Jochem H. Smit, Athina G. Portaliou, Katerina E. Chatzi, Spyridoula Karamanou, Ana-Nicoleta Bondar, Giorgos Gouridis, and Anastassios Economou. 2021. “A Nexus of Intrinsic Dynamics Underlies Translocase Priming.” *Structure* 29 (8): 846–858.e7. https://doi.org/10.1016/j.str.2021.03.015.

Masson, Glenn R., John E. Burke, Natalie G. Ahn, Ganesh S. Anand, Christoph Borchers, Sébastien Brier, George M. Bou-Assaf, et al. 2019. “Recommendations for Performing, Interpreting and Reporting Hydrogen Deuterium Exchange Mass Spectrometry (HDX-MS) Experiments.” *Nature Methods* 16 (7): 595–602. https://doi.org/10.1038/s41592-019-0459-y.

Puchała, Weronika, Michał Burdukiewicz, Michał Kistowski, Katarzyna A. Dąbrowska, Aleksandra E. Badaczewska-Dawid, Dominik Cysewski, and Michał Dadlez. 2020. “HaDeX: An R Package and Web-Server for Analysis of Data from Hydrogen-Deuterium Exchange Mass Spectrometry Experiments.” *Bioinformatics (Oxford, England)* 36 (16): 4516–18. https://doi.org/10.1093/bioinformatics/btaa587.

Weis, David D. 2021. “Recommendations for the Propagation of Uncertainty in Hydrogen Exchange-Mass Spectrometric Measurements.” *Journal of the American Society for Mass Spectrometry* 32 (7): 1610–17. https://doi.org/10.1021/jasms.0c00475.
